# Knockdown of deleterious miRNA in progenitor cell–derived small extracellular vesicles enhances tissue repair in myocardial infarction

**DOI:** 10.1126/sciadv.abo4616

**Published:** 2023-03-03

**Authors:** Hyun-Ji Park, Jessica R. Hoffman, Milton E. Brown, Sruti Bheri, Olga Brazhkina, Young Hoon Son, Michael E. Davis

**Affiliations:** ^1^ Wallace H. Coulter Department of Biomedical Engineering, Emory University School of Medicine and Georgia Institute of Technology, Atlanta, GA 30322, USA.; ^2^ Department of Molecular Science and Technology, Ajou University, Suwon 16499, South Korea.; ^3^ Molecular and Systems Pharmacology Graduate Training Program, Graduate Division of Biological and Biomedical Sciences, Laney Graduate School, Emory University, Atlanta, GA 30322, USA.; ^4^ Children's Heart Research and Outcomes (HeRO) Center, Children's Healthcare of Atlanta and Emory University, Atlanta, GA 30322, USA.

## Abstract

Small extracellular vesicles (sEVs) play a critical role in cardiac cell therapy by delivering molecular cargo and mediating cellular signaling. Among sEV cargo molecule types, microRNA (miRNA) is particularly potent and highly heterogeneous. However, not all miRNAs in sEV are beneficial. Two previous studies using computational modeling identified miR-192-5p and miR-432-5p as potentially deleterious in cardiac function and repair. Here, we show that knocking down miR-192-5p and miR-432-5p in cardiac c-kit^+^ cell (CPC)–derived sEVs enhances the therapeutic capabilities of sEVs in vitro and in a rat in vivo model of cardiac ischemia reperfusion. miR-192-5p– and miR-432-5p–depleted CPC-sEVs enhance cardiac function by reducing fibrosis and necrotic inflammatory responses. miR-192-5p–depleted CPC-sEVs also enhance mesenchymal stromal cell–like cell mobilization. Knocking down deleterious miRNAs from sEV could be a promising therapeutic strategy for treatment of chronic myocardial infarction.

## Teaser

Bioinformatics identifies specific deleterious miRNAs in cell-derived sEVs and depletion of these miRNA enhance cardiac function.

## INTRODUCTION

Ischemic heart diseases, including myocardial infarction (MI), are a leading cause of global death with an estimated 17.9 million deaths in 2019 alone (*1*). MI results in irreversible damage to the myocardium and leads to various complications as a result of impaired cardiovascular function (*2*). The lack of oxygen supply induces death of the ischemic tissue and eventually leads to heart failure. Now, the only cure for heart failure is transplantation, which is limited by donor availability and rejection. Over the past two decades, cell therapy has emerged as a potential approach for repair of the myocardium. Several adult stem and progenitor cell types have previously been tested in patients with MI, cardiomyopathies, and cardiac arrythmia (*3*–*7*). Recent evidence indicates that cell therapy showed beneficial effects following MI, albeit with modest benefits (*8*–*11*).

Originally, it was thought that cell therapy worked through direct replacement of the myocardium via regeneration. A number of studies have demonstrated compelling evidence that this does not occur (*12*, *13*). More recent work has demonstrated that cardiac cell therapy functions via paracrine signaling, or the release of soluble factors (*14*, *15*). In particular, transplanted cells release extracellular vesicles (EVs) loaded with various potentially beneficial cargo molecules. Small EVs (sEVs), including exosomes, are between 30 and 150 nm and are formed via exocytosis (*16*). During sEV biogenesis, cells load nucleic acids, proteins, and other molecules into the vesicle and release the vesicle into the extracellular space, where it may be internalized by neighboring cells. In this manner, sEVs play a critical role in cell therapy by delivering molecular cargo and mediating intercellular or interorgan signaling (*17*). The beneficial effects of sEV have been largely attributed to cargo microRNA (miRNA): short noncoding RNA that binds mRNA and induces degradation (*18*). For example, previous work demonstrated that sEV miRNA-21 exerts an antiapoptotic effect and restores cardiac function after MI (*19*).

Furthermore, sEV miRNA cargo is highly variable, and RNA sequencing (RNA-seq) and computational approaches are used to understand the importance of certain miRNAs under various conditions. Our group and others have shown that partial least squares regression (PLSR) modeling can be used to understand how sEV miRNA cargo covaries with functional effects (*20*–*22*). By using previously published data from our group, we identified the most important sEV miRNAs for angiogenesis, fibrosis, and left ventricular ejection fraction (EF) responses by calculating and ranking variable importance in projection (VIP) scores for each miRNA (*20*, *21*). From both studies’ top 50 sEV miRNA VIP lists, we identified miR-192-5p and miR-432-5p as potentially deleterious to cardiac repair.

In this study, we demonstrate that knockdown of sEV miR-192-5p or miR-432-5p, identified from previous computational modeling, enhances the therapeutic potential of cardiac c-kit^+^ cell (CPC)–derived sEVs. sEVs with reduced miR-192-5p or miR-432-5p levels can efficiently reduce fibrosis, regulate macrophage plasticity in ischemic hearts, and recruit bone marrow–derived mesenchymal stromal cells (MSCs). Moreover, intramuscular delivery of the modified sEVs improved cardiac function in a rat model of coronary artery ligation. Last, we identified the molecular mechanisms of miR-192-5p– and miR-432-5p–depleted sEVs on inflamed cardiac fibroblasts and immune cells with RNA-seq. Our results highlight the potential of miRNA inhibition in sEVs, which could be an overlooked strategy for the treatment of cardiac disease.

## RESULTS

### Target miRNAs, miR-192-5p and miR-432-5p, are selectively depleted in sEVs

**Fig. 1.**
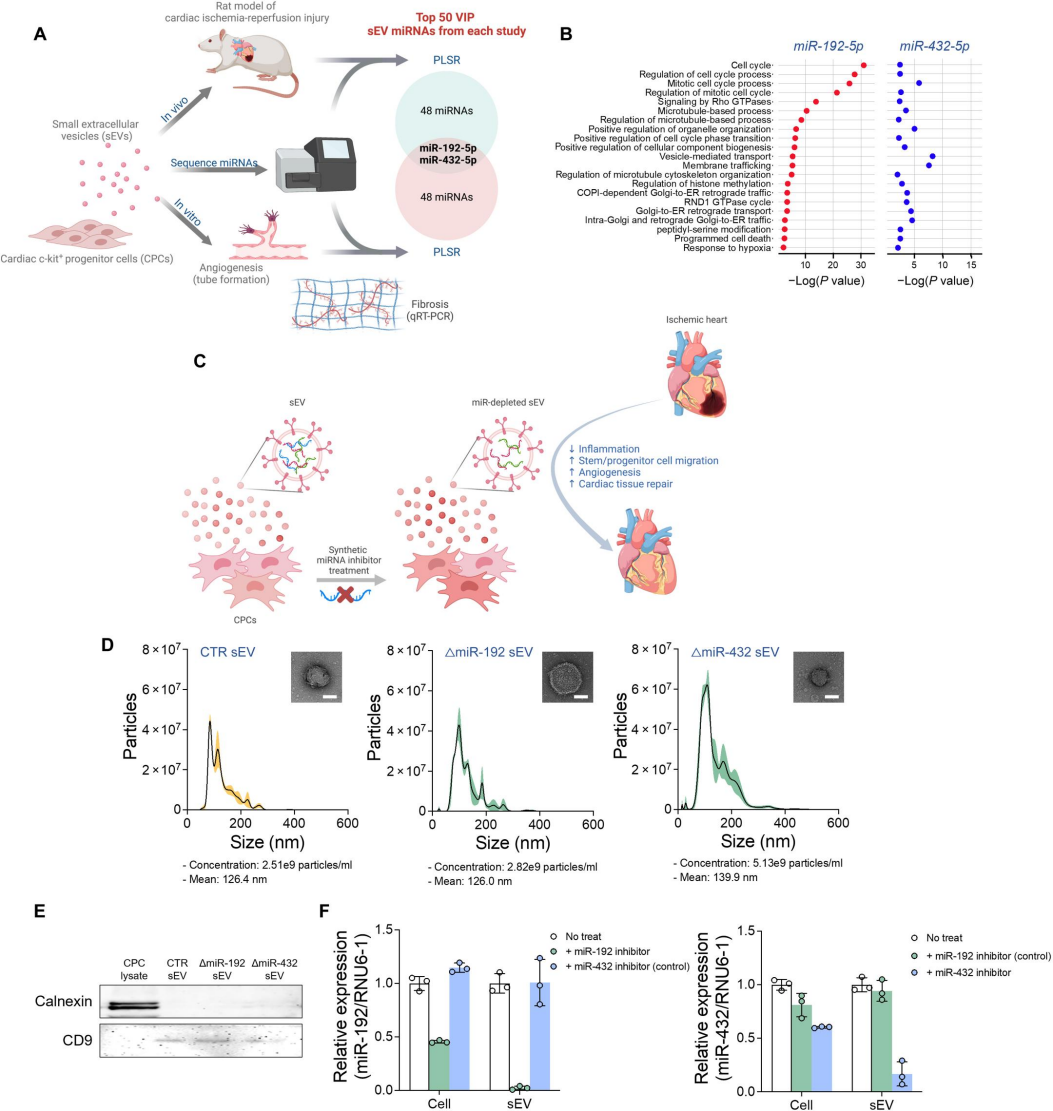
Human CPC-sEV composition and miRNA content were not altered by miR-192-5p and miR-432-5p depletion. (**A**) Venn diagram of top 50 most influential miRNAs identified from previously published studies (*20*, *21*). miR-192-5p and miR-432-5p were identified from the intersection of the two independent studies using computational models to link CPC-sEV miRNA to cardiac repair in in vitro and in vivo models. (**B**) Biological pathway enrichment of miR-192-5p and miR-432-5p gene targets. (**C**) Schematic illustration of miRNA knockdown in sEVs produced from synthetic miRNA inhibitor–treated CPCs. (**D**) Nanoparticle tracking analysis and TEM imaging of sEVs with and without inhibitor treatment. Inserted images placed in the top right of each graph showed the corresponding sEVs. (**E**) Western blot of tetraspanin 9 and calnexin from CPC and sEV lysate, with and without inhibitor treatment. (**F**) Relative expression of miR-192-5p and miR-432-5p from CPCs and their sEVs with and without inhibitor treatment.

In two previous studies, our group used computational modeling to identify CPC-sEV miRNAs that contributed the most to both in vitro and in vivo cardiac repair models (*20*, *21*). Among each study’s top 50 miRNAs highly connected to the processes of angiogenesis, fibrosis, and improvement of left ventricular EF, we determined that the two miRNAs, miR-192-5p and miR-432-5p, were deemed important in both studies (Fig. 1 and table S1). To further understand the biological context of miR-192-5p and miR-432-5p, we then used miRTarBase to determine their known gene targets. We found 1057 and 174 putative gene targets of miR-192-5p and miR-432-5p, respectively, 26 and 3 of which had been experimentally validated by at least three methods (table S2). Furthermore, the gene ontology (GO) term enrichment showed that miR-192-5p targets are related to cell cycle, proliferation, apoptosis, immune responses, microtubule process, and vascular development, whereas miR-432-5p targets are related to vesicular transport, metabolism, cell proliferation, and adhesion (Fig. 1B and fig. S1). Our previous studies suggested that miR-192-5p and miR-432-5p are potentially deleterious to cardiac repair; thus, we knocked down the expression of these two miRNAs in sEVs and further studied their mechanisms and therapeutic potential through RNA-seq and experimental methods using in vitro and in vivo models.

We depleted mature miRNA expression in CPC-sEVs by treating parent CPCs with either a synthetic miR-192-5p or miR-432-5p inhibitor (Fig. 1C). sEVs were isolated by sequential ultracentrifugation from the supernatant of CPCs grown in serum-free medium for 24 hours after miRNA inhibitor treatment. Preparations had a median size (120 to 140 nm), consistent with sEVs (Fig. 1D). As expected, the sEV marker tetraspanin CD9 was detected in sEVs, but the endoplasmic reticulum protein calnexin was not present in these preparations (Fig. 1E). We evaluated relative miRNA expression levels in sEVs and cells by quantitative reverse transcription polymerase chain reaction (qRT-PCR), normalizing to U6 small nuclear 1 (RNU6-1) (Fig. 1F). Although the inhibitors were treated to the cells and not directly to sEVs, the miRNA levels in sEVs (<4% of miR-192-5p expression and <20% of miR-432-5p expression compared to sEVs without inhibitor treatment) were much lower than those in cells (<50% of miR-192-5p expression and <65% of miR-432-5p expression compared to cells without inhibitor treatment), indicating that the intracellular delivery of the inhibitor reduces miR-192-5p and miR-432-5p levels in subsequently released sEVs.

### miR-192-5p– or miR-432-5p–depleted sEVs improve in vitro therapeutic efficacy

We sought to test whether miR-192-5p– or miR-432-5p–depleted sEVs could improve in vitro outcomes related to cardiac repair. GO enrichment analyses of miRNA target genes suggested that depletion of miR-192-5p has more anti-inflammatory and proangiogenic potential, whereas depletion of miR-432-5p has a more antifibrotic potential (Fig. 1B and fig. S1).

**Fig. 2.**
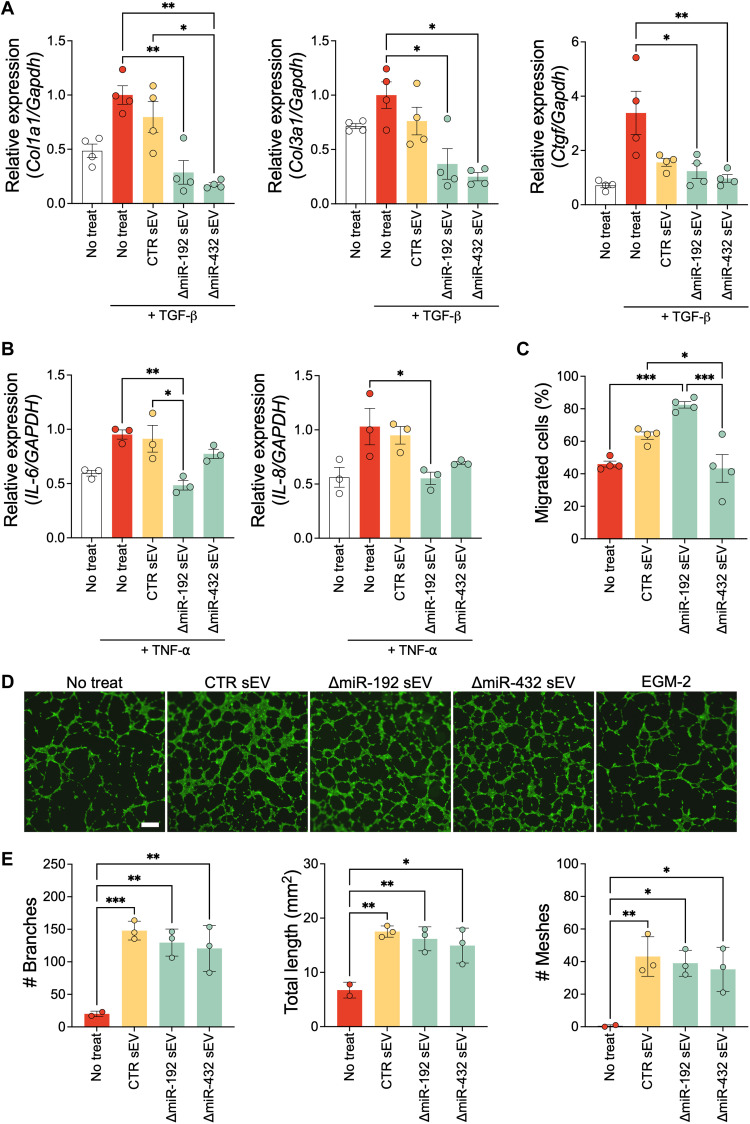
CPC-sEVs affect biological functions of various cardiac cells in vitro. (**A**) Fibrotic gene (*Col1a1*, *Col3a1*, and *Ctgf*) expression in RCFs 24 hours after sEV treatment. RCFs were activated with TGF-β 12 hours after sEV treatments. (**B**) Proinflammatory gene (*IL-6* and *IL-8*) expression in THP-1 monocytes 24 hours after sEV treatment. THP-1 monocytes were activated by TNF-α 12 hours after sEV treatments. (**C**) Percentage of migrated hMSCs over total cells seeded 24 hours after sEV treatment. (**D**) Representative images of rat CECs on Matrigel. (**E**) Quantification of number of branches, total length of the tube, and number of meshes measured by Fiji software. *P < 0.05, **P < 0.01, and ***P < 0.001.

To investigate the effects of miR-192-5p–depleted sEVs (ΔmiR-192 sEV) or miR-432-5p–depleted sEVs (ΔmiR-432 sEV), we treated various cardiac cells with the altered sEVs. First, the antifibrotic effect of ΔmiR-192 sEV and ΔmiR-432 sEV was investigated on rat cardiac fibroblasts (RCFs). RCFs were pretreated with sEVs and activated by transforming growth factor–β (TGF-β) to mimic the fibrotic environment of tissue injury (*23*). sEV pretreatment significantly reduced *Col1a1*, *Col3a1*, and *Ctgf* expressions in TGF-β–stimulated RCFs compared to those without sEV pretreatment (Fig. 2A). Furthermore, while there were no differences in untreated and control (CTR) sEV–treated RCFs, ΔmiR-192 sEV and ΔmiR-432 sEV treatment significantly reduced *Col1a1*, *Col3a1*, and *Ctgf* expression in inflamed RCFs. In particular, miR-432 depletion significantly decreased *Col1a1* expression compared to CPC-derived sEVs (CTR sEV). In addition, we examined the anti-inflammatory effect of altered sEVs on monocytes in vitro. Overall, inflammatory gene expression (*IL-6* and *IL-8*) in tumor necrosis factor–α (TNF-α)–induced human monocytes was decreased by altered sEV treatment (Fig. 2B). Specifically, ΔmiR-192 sEV treatment, but not ΔmiR-432 sEV treatment, significantly decreased *IL-6* expression compared to no sEV treatment and CTR sEV treatment groups (0.48 ± 0.08, 0.77 ± 0.07, 0.95 ± 0.07, and 0.91 ± 0.21, respectively). Notably, these in vitro experimental results reflect our GO enrichment analyses: ΔmiR-192 sEV treatment significantly reduced proinflammatory cytokine expression, whereas ΔmiR-432 sEV treatment more effectively reduced fibrotic gene expression.

In addition to investigating altered sEV anti-inflammatory and antifibrotic effects, we also examined sEV effects on MSC migration. MSCs contribute to angiogenesis and tissue repair by recruiting endogenous epithelial cells and regulating inflammatory cells (*24*, *25*). To investigate the effect of sEV on MSC mobilization, we performed an in vitro cell migration assay using a Boyden chamber system (Fig. 2C). We generated an sEV gradient with 8-μm transwell insert (MSCs seeded on the apical side and 20 μM sEV in the basolateral compartment) and measured MSC migration to the basolateral side of the membrane. We found that ΔmiR-192 sEV, but not CTR sEV and ΔmiR-432 sEV, facilitated the cell migration (45.97 ± 3.62%, 63.46 ± 4.58%, 82.40 ± 4.00%, and 43.30 ± 17.02% in No treat, CTR sEV, ΔmiR-192 sEV, and ΔmiR-432 sEV groups, respectively) (Fig. 2C).

Last, the angiogenic effects of the different sEVs on rat cardiac endothelial cells (CECs) were investigated using a tube formation assay. All sEV treatment groups significantly enhanced CEC total tube length, number of branches, and number of meshes (Fig. 2, D and E). However, there were no notable improvement in tube formation in altered sEV groups, in comparison to CTR sEV.

### RNA-seq reveals the downstream effects of miR depleted sEVs

**Fig. 3.**
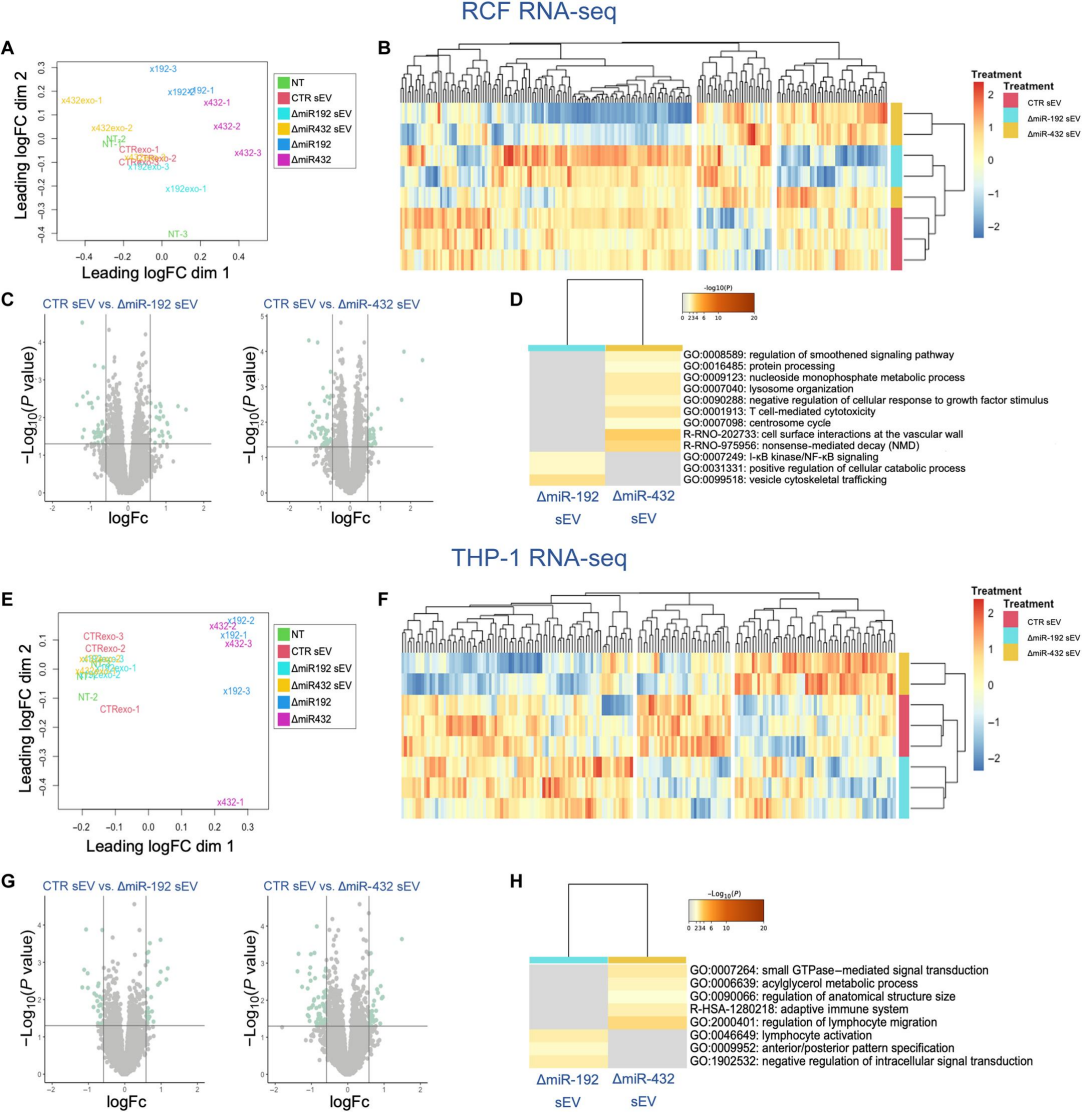
RNA expression in RCF and THP-1 cells was altered by sEV treatment. Multidimensional scaling plot of (**A**) RCFs and (**E**) THP-1 monocytes with and without sEV treatment. Heatmap of differentially expressed RNAs in groups of CTR sEV–, ΔmiR-192 sEV–, and ΔmiR-432 sEV–treated (**B**) RCFs and (**F**) THP-1 monocytes. Volcano plot of differentially expressed RNAs in CTR sEV versus ΔmiR-192 sEV, CTR sEV versus ΔmiR-432 sEV (**C**) RCFs and (**G**) THP-1 monocytes. GO pathway enriched terms from differentially expressed RNAs in (**D**) RCFs and (**H**) THP-1 monocytes.

To understand the downstream effects and potential mechanisms of action of ΔmiR-192 and ΔmiR-432 sEVs, we treated fibroblasts and monocytes with sEVs or miRNA inhibitors and measured recipient cell gene expression with RNA-seq (fig. S2). Specifically, for RCFs, we pretreated with sEVs or miRNA inhibitors, stimulated the cells with TGF-β to induce fibrosis, and examined differentially expressed genes (DEGs). First, we determined with multidimensionally scaling that RCFs treated with sEVs clustered separately from cells treated only with the miR inhibitors, with samples separating across the first dimension (Fig. 3A). Furthermore, we found that RCFs treated with CTRsEVs cluster most similarly with cells treated with ΔmiR-192 sEVs. Differential expression analysis of RCFs revealed that few genes were differentially expressed in cells pretreated with the sEV groups (55 genes CTR sEV versus ΔmiR-192 sEV, 70 genes CTR sEV versus ΔmiR-432 sEV) (Fig. 3, B and C). Furthermore, genes up-regulated in ΔmiR-192 sEVs, as compared to CTR sEV, were enriched in nuclear factor κ-light-chain enhancer of activated B cells (NF-κB) signaling and vesicle cytoskeleton trafficking. Pathway analysis of genes up-regulated in ΔmiR-432, as compared to CTR sEV, is involved in smoothened signaling pathway and cell surface interactions at the vascular wall (Fig. 3D).

In addition, we pretreated THP-1 cells with sEVs or miR inhibitors, stimulated the cells with TNF-α to induce inflammation, and examined downstream DEGs with RNA-seq. Similar to RCFs, THP-1 cells treated with sEVs clustered separately from cells treated with the miRNA inhibitors (Fig. 3E). Analysis of THP-1 cells revealed that ~0.5% of genes were differentially expressed between sEV groups (50 genes CTR sEVs versus ΔmiR-192 sEV, 91 genes CTR sEVs versus ΔmiR-432 sEV) (Fig. 3, F and G). Pathway analysis of these DEGs in THP-1 cells revealed enrichment of immune processes including lymphocyte activation in ΔmiR-192 sEV, and adaptive immune system and regulation of lymphocyte migration in ΔmiR-432 sEV (Fig. 3H). Overall, given our GO analysis of miR-192 and miR-432 gene targets (Fig. 1B and fig. S1) and in vitro results (Fig. 2), we expected to see that ΔmiR-192 sEVs affect inflammatory pathways and that ΔmiR-432 sEVs affect fibrotic pathways.

### Echo-guided injection of altered sEVs improves left ventricular EF and FS in the rat model of cardiac IR

**Fig. 4.**
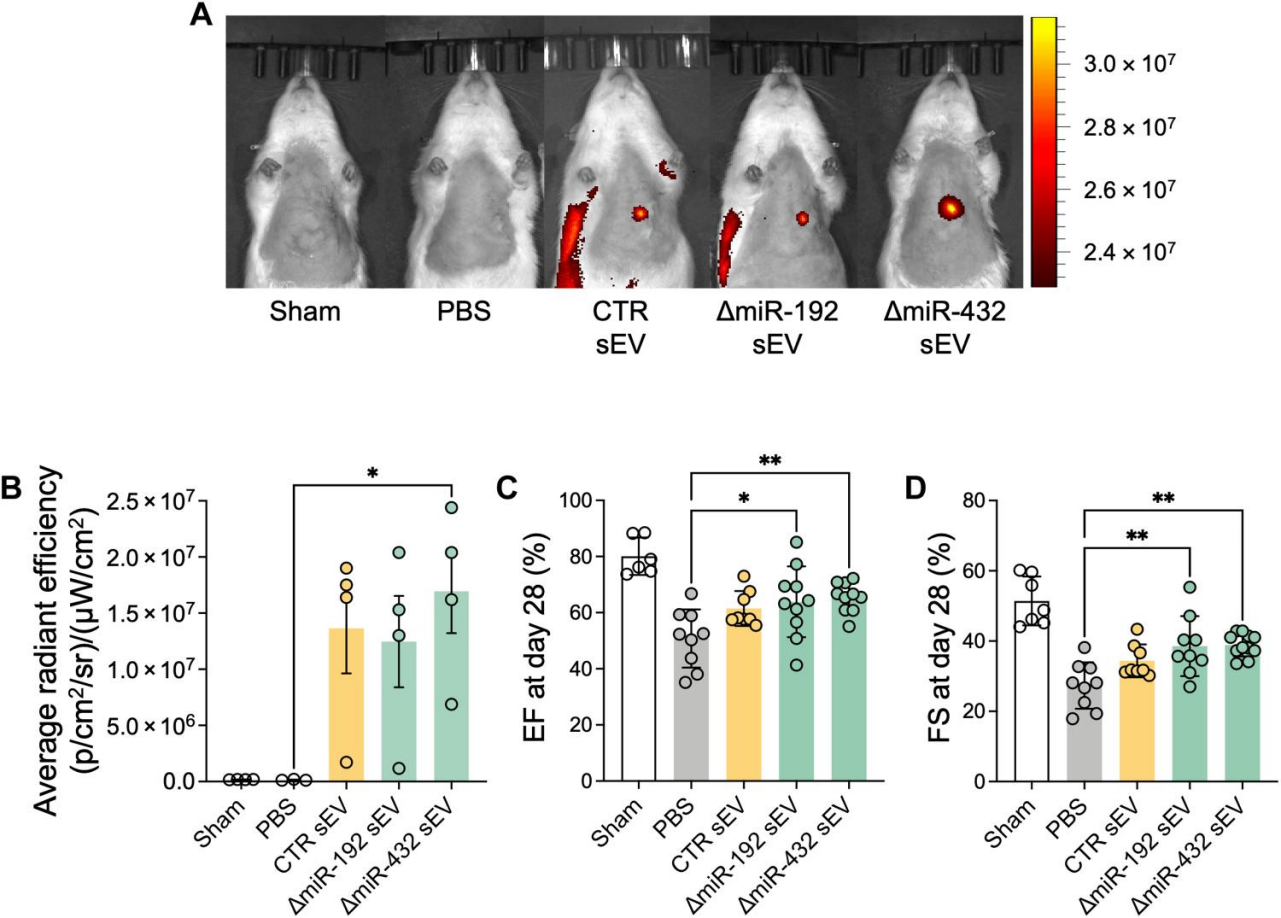
Cardiac function was improved after echo-guided sEV administration to ischemic heart. (**A**) Representative images of in vivo live imaging of sEVs after echo-guided intramyocardial injection into ischemic heart. Fluorescent images of rats shown 7 days after echo-guided myocardial injection of sEVs (5 μg/kg). (**B**) Quantification of average radiant efficiency [(p/s/cm^2^)/(sr/μW/cm^2^)] from the rat heart. (**C**) Left ventricular EF and (**D**) left ventricular FS measured at day 28 after sEV injection. *P < 0.05 and **P < 0.01.

To determine the reparative effects of the modified sEVs, we directly injected sEVs to the ischemic myocardium of male Sprague-Dawley rats 2 weeks following 30-min ischemia-reperfusion (IR) of the left anterior descending coronary artery. All rats received baseline echocardiography and then were randomized to treatment groups 2 weeks following injury in a double-blinded manner. To test sEV retention in the myocardium, DiR-stained sEVs (5 μg/kg) were delivered via echo-guided injection intramuscularly to rats 2 weeks after IR surgery. Radiant efficiency data from the IVIS imaging showed accumulation of sEVs in the heart for all sEV groups at day 7 after sEV injection (Fig. 4, A and B). In addition, there were no notable differences in retention between sEV groups for 14 days (fig. S3).

The therapeutic effect of ΔmiR-192 and ΔmiR-432 sEV injection in IR rats was compared with CTR sEV injection by assessing cardiac function. Twenty-eight days after injection, sEV injection led to significant improvement of left ventricular EF and fractional shortening (FS), compared to phosphate-buffered saline (PBS) control (Fig. 4, C and D). ΔmiR-192 and ΔmiR-432 sEV administration improved EF and FS compared to no treatment groups (PBS group). However, there were no statistically significant improvement in altered sEV groups compared to CTR sEV group.

### sEVs improve endogenous stem cell mobilization and attenuate necrotic inflammatory responses after the early onset of cardiac ischemia

**Fig. 5.**
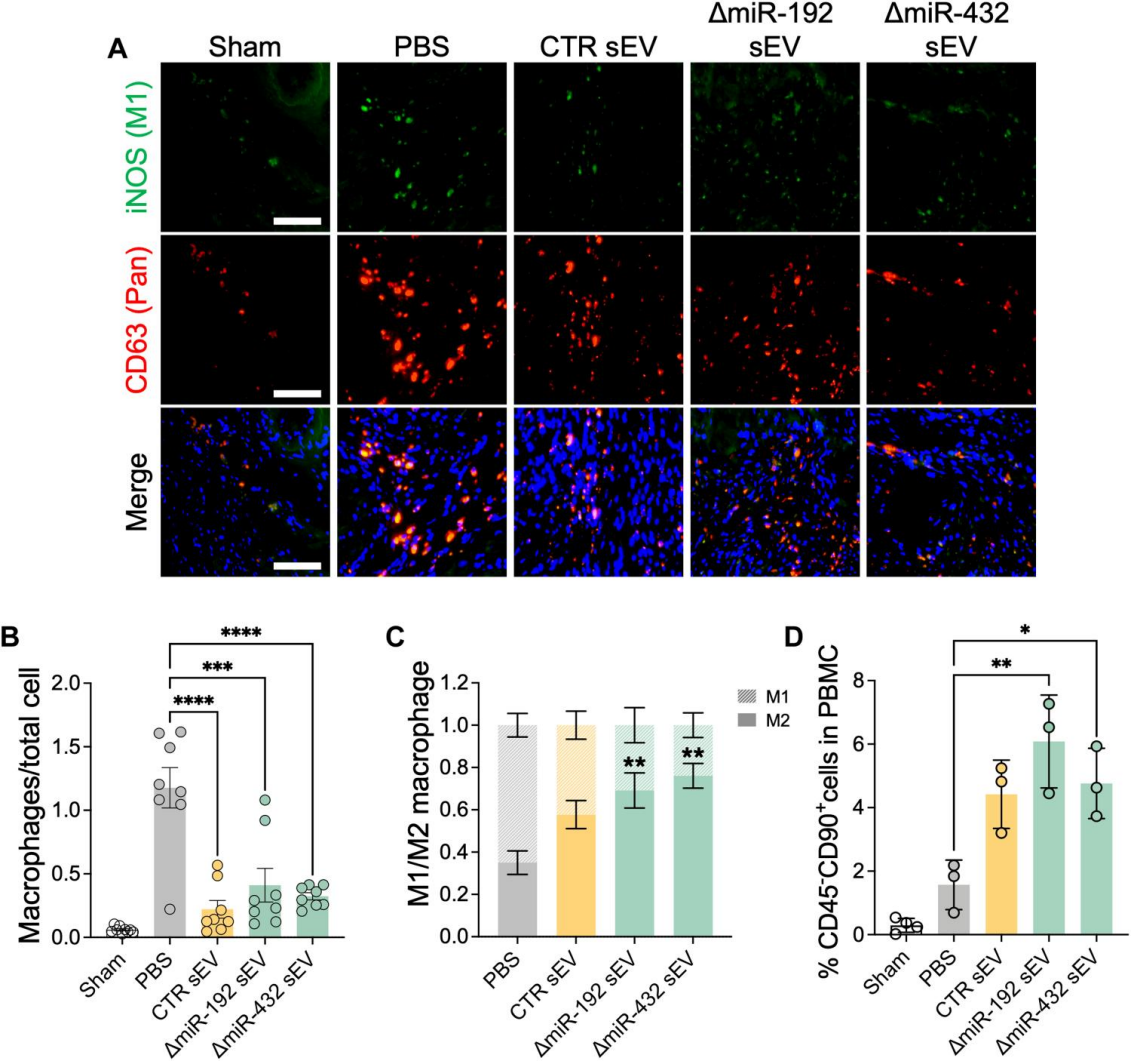
Immunomodulatory effect of sEV attenuated inflammatory responses in cardiac IR rats. (**A**) Immunohistochemical staining of ischemic hearts to detect CD63^+^ total macrophages and iNOS^+^CD63^+^ M1-type macrophages (CD63 for red and iNOS for green staining). Scale bars, 100 μm. (**B**) Quantification of the total number of CD63^+^ macrophages. ***P < 0.001 and ****P < 0.0001. (**C**) Quantification of the ratio of iNOS^−^CD63^+^ cells (M2 type) to iNOS^+^CD63^+^ cells (M1 type) (*n* = 8). **P < 0.01 versus M2 macrophage proportion in the PBS group. (**D**) Blood circulating CD45^−^CD90^+^ cell population (MSC) in total PBMCs from cardiac IR rats. *P < 0.05, **P < 0.01, and ***P < 0.001.

To investigate immunomodulatory effects of sEVs, sEVs were injected to cardiac IR rats, and the local inflammatory effect through M2-like macrophage activation in ischemic cardiac muscle was measured after 7 days. Notably, M1-type macrophages secrete proinflammatory cytokines and sterilize damaged tissues, whereas M2-type macrophages secrete anti-inflammatory cytokines and facilitate tissue healing (*26*). Thus, the ratio of M2/M1 macrophages can reveal the stage of tissue repair in the injured tissues. In the normal muscle without ischemic injury (Sham group), few macrophages were observed in muscle tissues (Fig. 5A). In contrast, ischemia substantially promoted infiltration of macrophages between muscle fibers (PBS group). However, injection of sEVs reduced immune cell infiltration, and ΔmiR-192 sEV and ΔmiR-432 sEV treatment showed significant numbers of M2-like macrophage phenotype (iNOS^−^CD63^+^ cells) accounting for around 70% of infiltrated macrophages (69.1 ± 20.4% in the ΔmiR-192 sEV group and 76.1 ± 14.2% in the ΔmiR-432 sEV group) (Fig. 5, B and C). In addition, we further evaluated inflammatory responses of sEVs in early onset of cardiac ischemia using histological analyses (fig. S5). The expressions of TNF-α and reactive oxygen species (ROS) in the left ventricular wall were decreased by sEV treatment, which further decreased to the Sham level by ΔmiR-192 and ΔmiR-432 sEV treatment (29.12 ± 12.83% TNF-α–positive area and 8.36 ± 1.47% ROS-positive area in the PBS group, 14.64 ± 5.27% TNF-α–positive area and 4.42 ± 1.96% ROS-positive area in the CTR sEV group, 4.02 ± 3.01% TNF-α–positive area and 2.17 ± 0.74% ROS-positive area in the ΔmiR-192 sEV group, 8.48 ± 7.46% TNF-α–positive area and 3.84 ± 2.04% ROS-positive area in the ΔmiR-432 TNF-α and sEV group, and 0.98 ± 1.03% TNF-α–positive area and 2.05 ± 2.50% ROS-positive area in the Sham group) (fig. S5, B and C). Overall, these results indicate that miR-192-5p and miR-432-5p depletion in sEVs can reduce local inflammation by modulating macrophage infiltration and selective activation and also prevent tissue necrosis in cardiac ischemia.

In addition to promoting M2-like macrophages into ischemic cardiac muscle, sEVs could exert their tissue healing effects by increasing stromal cell mobilization from bone marrow to blood circulation during early time points of cardiac remodeling. To determine the effect of sEVs on stem cell mobilization from bone marrow to blood circulation in IR rats, the population of circulating MSCs in peripheral blood mononuclear cells (PBMCs) was analyzed by flow cytometry 7 days after sEV injection. We found no CD45^−^CD90^+^ MSCs in our Sham group. Similar to our in vitro findings, sEV treatment promoted MSC mobilization in rats with IR injury, with ΔmiR-192 sEV treatment increasing PBMC MSC to the greatest extent (Fig. 5D). Overall, our data suggest that ΔmiR-192 sEVs can augment endogenous MSC mobilization in IR rats.

### ΔmiR-192 and ΔmiR-432 sEVs promote angiogenesis and prevent hypertrophy

**Fig. 6.**
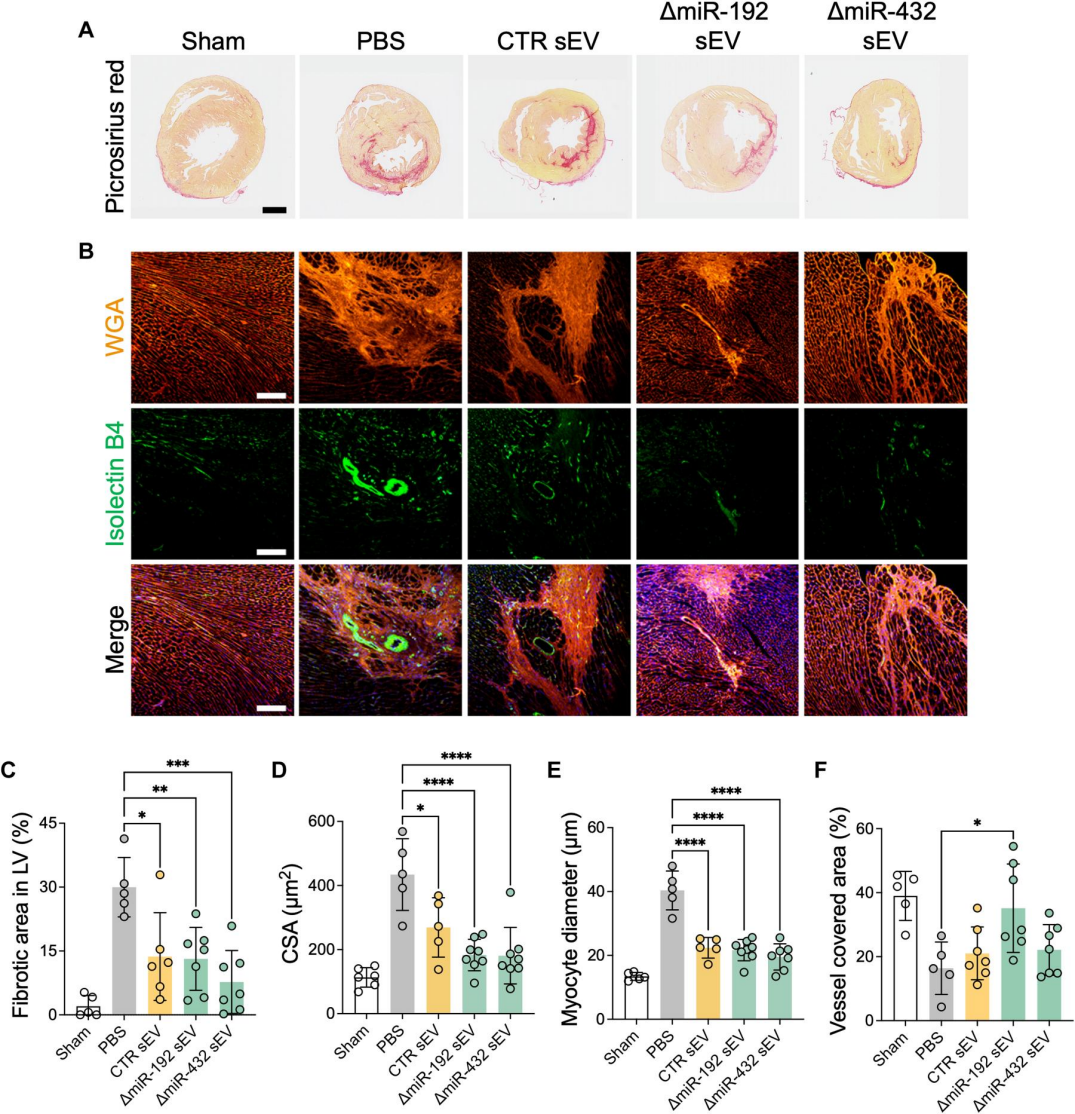
Histological analyses showed the improved ischemic heart function by sEV injection in cardiac IR rats 28 days after treatment. (**A**) Representative images of picrosirius red–stained heart 28 days after sEV injection. Scale bar, 1 mm. (**B**) WGA-positive hypertrophy and isolectin B4 (IB4)–positive vessels in ischemic muscles 28 days after sEV injection. Scale bars, 200 μm. (**C**) Fibrotic area in left ventricular (LV) muscle quantified from picrosirius red–stained images. (**D**) Cross-sectional area (CSA) and (**E**) myocyte diameter measured from WGA-stained images. (**F**) Vessel covered area measured from *Isolectin B4*–stained images. *P < 0.05, **P < 0.01, ***P < 0.001, and ***P < 0.0001.

sEVs produced from CPCs or other stem/progenitor cells are known to have a therapeutic potential in cardiac ischemia (*20*, *21*). In addition, our in vitro and short-term in vivo experimental results showed enhanced potential of ΔmiR-192 and ΔmiR-432 sEVs for cardiac repair in chronic cardiac ischemia by immunomodulation and MSC mobilization effects (Figs. 2 and 5). Histological analyses of picrosirius red staining with ischemic heart 28 days after treatment revealed that sEV injection prevented muscle degeneration and fibrosis in the left ventricular wall more than twofold than the PBS group (29.99 ± 6.98% in the PBS group, 13.69 ± 10.30% in the CTR sEV group, 13.15 ± 7.39% in the ΔmiR-192 sEV group, and 7.72 ± 7.40% in the ΔmiR-432 sEV group) (Fig. 6, A and C). Notably, this result correlates with our in vitro data (Fig. 2A). Overall, the rapid restoration of inflammation and MSC mobilization by sEV therapy in the early stage of chronic ischemia progress (within 7 days) effectively prevented cardiac dysfunction by abrogating fibrotic events and muscle degeneration.

We further evaluated the effect of sEV injection on left ventricular repair using histological analyses. First, the ventricular hypertrophy was decreased in the left ventricular wall treated by sEVs, as confirmed by immunohistochemical staining of wheat germ agglutinin (WGA) (Fig. 6B). The cross-sectional area (CSA) and myocyte diameter were also decreased by sEV treatment, which further decreased to the Sham level by ΔmiR-192 and ΔmiR-432 sEV treatment (Fig. 6, D and E). In addition, the densities of isolectin B4 (IB4)–positive vessel were highest in ΔmiR-192 sEV group, indicating that sEVs, especially with miR-192-5p depletion, facilitated blood vessel formation in ischemic muscle (16.38 ± 8.20% in the PBS group, 21.03 ± 8.25% in the CTR sEV group, 35.14 ± 13.81% in the ΔmiR-192 sEV group, 22.13 ± 7.90% in the ΔmiR-432 sEV group, and 38.98 ± 7.67% in the Sham group) (Fig. 6, B and F). These results demonstrate that altered sEVs have increased therapeutic potential for treating chronic cardiac ischemia and can significantly improve tissue repair by decreasing hypertension and increasing angiogenesis.

## DISCUSSION

In this study, we investigated the inhibition of computationally determined, deleterious miRNAs in sEVs to treat chronic MI. In two previous studies, our group determined with computational approach, PLSR, that elevated miR-192-5p and miR-432-5p covary with reduced left ventricular EF, antiangiogenic, and profibrotic responses in vitro and in vivo. Therefore, we investigated CPC-derived sEVs with miR-192-5p or miR-432-5p knockdown to treat chronic MI (Fig. 1C). To do so, we treated CPCs with synthetic miRNA inhibitors, double-stranded RNA, and confirmed knocked down in subsequently released and isolated sEVs. Not only did miRNA inhibition in CPCs confer reduced expression of miR-192-5p and miR-432-5p in sEVs (Fig. 1F) but also inhibitor treatment did not alter sEV production, size, or sEV membrane proteins (Fig. 1, D and E), suggesting cellular miRNA inhibitor treatment as an effective means to alter sEV RNA cargo. Our results demonstrate that miR-192-5p– and miR-432-5p–depleted sEVs are antifibrotic, anti-inflammatory, and proangiogenic in vitro and improve cardiac function following chronic MI in rats.

Our group has studied CPC-sEV miRNA content with computational modeling in several studies (*20*, *21*); however, we have never investigated individual miRNAs identified from previous studies for the purpose of modifying sEVs and improving cardiac therapeutic potential. miR-192-5p and miR-432-5p were identified as important and potentially deleterious in two previous studies from our group: Agarwal *et al.* (*20*) and Trac *et al.* (*21*). In these studies, our group sequenced miRNAs in CPC-derived sEVs and constructed PLSR models with the sequencing data and experimental outcomes (sEV treatment effects on left ventricular EF in an in vivo rat MI model, and fibrosis and angiogenesis assays in vitro). These models produced scores for each miRNA based on covariance to experimental outcomes, and we determined the top 50 most influential miRNAs in each study, either beneficial or deleterious. Both studies identified miR-192-5p and miR-432-5p as influential miRNAs, potentially important in cardiac repair (Fig. 1A).

miR-192-5p, a member of the miR-192 family, is known to be closely involved in cancer pathogenesis. It regulates oxidative stress (*27*), cell proliferation, apoptosis (*27*), and immune reactions (*28*) through involvement in several pathways including p53 and TGF-β (*29*, *30*). miR-192-5p is also a potential serum exosomal biomarker for heart failure patients as its expression was increased in dilated cardiomyopathy patients’ sera (*31*). Similar to miR-192-5p, miR-432-5p induces cardiac hypertrophy, but by binding to Toll-like receptor 4 (TLR4) in response to the up-regulation of long noncoding RNA, CASC15 (*32*). These reports support our hypothesis of two identified miRNAs, miR-192-5p and miR-432-5p, from computational approaches as deleterious to cardiac function and repair.

Under physiological conditions, miRNAs are encapsulated in EVs and internalized by neighboring cells, tissue, or organs. EVs are produced from various cells by extracellular membrane budding or exocytosis; thus, their membrane composition highly depends on the parental cell types. The tissue-specific membrane protein composition allows sEVs to have selective tropism based on their parental cell sources (*33*). A previous report showed that MSC-derived exosomes and monocyte-derived exosomes were selectively taken up by the cells that are in the same lineage as their parental cell types in an MSC/monocyte coculture system (*33*). The sEVs used in this study were produced by CPCs, cells isolated from cardiac biopsy from congenital heart patients; thus, these sEVs may exhibit beneficial cardiac-specific tropism.

Our results indicate that sEVs existed in the cardiac muscle for at least 7 days after injection (Fig. 4, A and B, and fig. S3). Seven-day retention was sufficient to induce macrophage propagation to M2 phase in ischemic myocardium. This effect is critical considering that immunomodulation in early time points of cardiac remodeling has been regarded as an important strategy to mitigate several cardiac complications including MI, arrhythmia, and aneurysms (*34*). Specifically, CPC-sEVs with miR-192-5p or miR-432-5p depletion showed enhanced immunomodulation (Figs. 2 and 5D and fig. S5), whereas only miR-192-5p–depleted CPC-sEVs improved MSC mobilization both in vitro (Fig. 2) and in vivo (Fig. 5D). Immunomodulatory effects of sEV were boosted by miR-192-5p depletion, leading to a significant decrease in mRNA levels of proinflammatory cytokines (*IL-6* and *IL-8*) (Fig. 2B). In addition, TNF-α–positive areas were decreased by sEV treatment, which further decreased to the Sham level by ΔmiR-192 and ΔmiR-432 sEV treatment (fig. S5, A and B). Among two altered sEVs, only miR-192-5p–depleted CPC-sEVs significantly suppressed ROS generation in ischemic cardiac muscle (fig. S5, A and C). Under the inflammatory condition, TNF-α triggers necrotic cell death by accelerating ROS generation (*35*). In addition, ROS induces necroptosis in response to TNF/TNF receptor 1 binding (*36*). Thus, in addition to expanded M2-like macrophages, sEV treatment, especially ΔmiR-192–depleted CPC-sEV administration, attenuated necrotic inflammatory responses by mitigating TNF-α and ROS expressions in ischemic cardiac muscles. Moreover, intramyocardial sEV injection enhanced M2-phase propagation of infiltrated macrophages for tissue healing, especially in the ΔmiR-432 sEV group (Fig. 5C). Thus, miR-192-5p and miR-432-5p depletion in sEVs beneficially alters the immune response during early time points of cardiac remodeling, thereby attenuating inflammation and promoting tissue healing after cardiac injury. The mobilization of MSC-like cells (CD45^−^CD90^+^) was also increased considerably by sEV treatments, especially in the ΔmiR-192 sEV group (Fig. 5D). Considering that MSCs are known to regulate macrophage activation and maturation, this finding supports our proinflammatory gene expression results in TNF-α–inflamed monocytes (Fig. 2B). In addition, it is important to note that we used one-fifth of the dose of sEVs as in our previous studies; thus, our data show that our modified sEVs may be more potent and warrant future studies on dose effects.

Ultimately, the sEV treatment substantially enhanced the restoration of cardiac function over sEV treatment in chronic ischemic heart, accompanied by improved left ventricular EF, FS, reduced fibrosis, muscle degeneration, and hypertrophy (Fig. 6). To further investigate the modified sEV mechanisms of action, we treated TNF-α–stimulated monocytes and TGF-β–stimulated fibroblasts with ΔmiR-192-5p sEV and ΔmiR-432-5p sEVs and performed RNA-seq of the recipient cells. RNA-seq results revealed that ΔmiR-192 sEV treatment affects NF-κB signaling and vesicle cytoskeletal trafficking in cardiac fibroblasts and lymphocyte activation in monocytes, whereas ΔmiR-432 sEV treatment activates biological processes such as smoothened signaling pathway and cell surface interaction in fibroblasts and adaptive immune system in monocytes (Fig. 3).

In the process of cardiac repair, the tissue remodeling in ischemic cardiac muscle increased myocyte CSA, which later decreased in all groups (fig. S6A); however, the myocyte diameter became enlarged in week 4 (fig. S6B). Note that excessive myocyte lengthening without a further change in CSA leads to chamber dilatation in the progression to cardiac failure (*37*), PBS group showed cellular alteration favorable to cardiac failure, whereas sEV treatment prevented chamber dilation and ultimately mitigated unfavorable cardiac remodeling (fig. S6C). Again, there is no evidence that miR-192-5p– and miR-432-5p–depleted CPC-sEVs have beneficial effect on tissue repair and cardiac function in animal model; however, miR-192-5p knockdown did show effective anti-inflammatory responses compared to other sEV treatment groups (Fig. 2B and fig. S5).

Although ΔmiR-192-5p or ΔmiR-432-5p sEVs showed beneficial effects on cellular assays, including antifibrosis, anti-inflammation, and MSC migration (Fig. 2), there were no statistical significance observed between altered sEVs and CTR sEVs in the rat model of cardiac IR (although only treatment with the depleted sEVs improved function, whereas CTR sEVs had no significant effect; Fig. 5). The method that we have used for specific miRNA depletion in this study was knockdown miR-192-5p or miR-432-5p expressions in CPC-sEVs <80%; however, knocking down these miRNAs in cells could alter the remaining cargo or have differential effects on the entire organ system. For example, one group reported that single miR-1-1 or miR-1-2 knockout is compensated by the negative feedback from remaining miR-1 in mice (*38*, *39*), resulting in mild ventricular dilation, whereas double knockout is lethal (*38*, *40*).

The in vitro fibrosis and inflammation models in this study were achieved by treating fibroblasts and monocytes with cytokines TNF-α and TGF-β (Fig. 2, A and B). While this is an acceptable method to replicate fibrotic and inflammatory pathological events, this model cannot fully recapitulate the complicated pathophysiological reactions of a myocardial ischemic event. We recognize the disparity of our in vitro and in vivo results: ΔmiR-192-5p and ΔmiR-432-5p sEVs improve in vitro, but not all in vivo, conditions, when compared to CTR sEVs. In addition, there might be more powerful deleterious miRNA candidates than miR-192-5p and miR-432-5p because this miR-192-5p was 39th and 44th and miR-432-5p was 41st and 38th of the top 50 miRNAs (table S1). Furthermore, we note that sEV miRNA is highly heterogeneous. Therefore, our single miRNA knockdown model may not be sufficient to improve sEV’s therapeutic potential. In this manner, multiple miRNA knockdowns could be used to overcome the differential therapeutic potential of single miRNA knockdown in in vivo models. Last, given that sEVs have complex membranes that exhibit favored tropism—as compared to synthetic vesicle alternatives—we believe that optimizing sEV cargo is a justifiable and effective approach.

Cardiac cell therapy, including CPCs, may have therapeutic efficacy for heart failure patients. There are several ongoing or completed clinical trials using autologous CPCs alone (CHILD, NCT03406884, phase 1 recruiting) or in combination with MSCs (CONCERT-HF, NCT02501811, phase 2 completed) (*8*) for the treatment of hypoplastic left heart syndrome and heart failure, respectively. Several reports suggest that CPC paracrine signaling, including the release of EVs, is the main source of cardiac cell therapy (*14*, *15*). However, results are variable and depend on several factors, including the patient’s age and cell culture condition. Previously, our group found that neonate CPCs and CPCs cultured under hypoxic conditions have enhanced therapeutic potential (*20*). In retrospect, we found that the levels of miR-192-5p and miR-432-5p are lower in the CPC exosomes cultured under hypoxic condition compared those under normoxic condition (fig. S4). Cells are highly sensitive to surrounding environmental changes; thus, the EV cargo content including miRNAs changes in response to the environment (*41*). The variability of EV cargo allows EVs to be reliable disease biomarkers, as well as therapeutic sources. Furthermore, genetic manipulation methods such as gene depletion or overexpression of certain miRNAs in sEV could enhance therapeutic potential of sEVs. Here, we used synthetic miRNA inhibitors in parent CPCs to specifically repress miR-192-5p and miR-432-5p expressions in the cells and sEVs.

In summary, CPC-derived altered sEV provides a cell-free system that leverages the therapeutic potential of CPCs while avoiding the risks associated with allogeneic cell transplantation. Under the proper conditions and patient variables, CPC-derived sEVs have cardiac reparative abilities: modulating cardiomyocyte function and inducing repair in the heart. However, sEV miRNA cargo is highly heterogeneous, and potentially not all molecules are beneficial. Therefore, optimization of sEV cargo, via the depletion of deleterious miRNAs from sEVs, may augment the therapeutic capabilities of CPC-derived sEVs. Here, we demonstrated the potential of miR-192-5p– and miR-432-5p–depleted CPC-derived sEVs to enhance cardiac function and MSC-like cell mobilization and to reduce fibrosis and inflammation. Considering that no treatment exists to fundamentally address chronic MI, miR-192-5p– or miR-432-5p–depleted CPC-sEV could be a promising therapeutic candidate for chronic MI patients.

## MATERIALS AND METHODS

### Cell culture and sEV isolation

CPCs were isolated from cardiac biopsies of congenital heart patients with a ventricular septal defect (VSD) under approval by the Institutional Review Board at Children’s Healthcare of Atlanta and Emory University, under protocol IRB00005500, as previously described (*42*). Briefly, tissue from the atrial appendage collected during the Glenn procedure was digested with collagenase II (1 mg/ml; Worthington Biochemical, NJ, USA), and c-kit^+^ cells were isolated through magnetic separation (Miltenyi Biotec, MD, USA). The cells that were isolated were subsequently identified as CPCs (c-kit positive, GATA positive, CD34 negative) through flow cytometry analysis. CPCs were cultured using Ham’s F-12 medium (Corning, MA, USA) supplemented with 10% fetal bovine serum (FBS; R&D Systems, MN, USA), 1% penicillin-streptomycin (Thermo Fisher Scientific, MA, USA), 1% l-glutamine, and fibroblast growth factor–2 (FGF-2; 4 ng/ml; Sigma-Aldrich, MO, USA). Cells were grown to 80 to 90% confluency to produce sEVs before collection. Thirty hours before sEV collection, cells were treated with the 1:1 mixture of Lipofectamine 2000 (Thermo Fisher Scientific) and MISSION Synthetic microRNA inhibitor (Sigma-Aldrich) for miRNA depletion under serum starvation in serum-free Ham’s F-12 medium. After 6 hours of miRNA inhibitor treatment, the medium was changed to fresh serum-free Ham’s F-12 medium and collected after 24 hours to isolate sEVs by sequential ultracentrifugation as previously described (*17*). All sEVs were resuspended in 300 μl of PBS and were quantified using nanoparticle tracking analysis on NanoSight NS300 (Malvern Panalytical, Malvern, UK).

### sEV characterization

sEV size and concentration were determined by nanoparticle tracking analysis. Briefly, sEV samples were diluted 1:10 in PBS, and 1 ml of sample was injected into NanoSight NS300 (Salisbury, UK) and three 60-s video acquisitions were captured per sample to analyze dynamic particle size and particle concentration by NanoSight NTA 3.4 software. Morphology and the size of EVs were also observed by transmission electron microscopy (TEM) imaging.

### Quantification of relative miRNA expressions in sEVs

To validate whether miR-192 and miR-432 in their sEVs were inhibited, the relative levels of miRNAs in CPC and their sEVs were measured by qRT-PCR analysis. Briefly, RNA was isolated from CPCs and their sEVs using the miRNeasy Micro Kit (Qiagen, Hilden, Germany), the concentration was measured using the Thermo NanoDrop One Microvolume UV-Vis Spectrophotometer (Thermo Fisher Scientific), RNA was reverse-transcribed into complementary DNA (cDNA) using the qScript microRNA cDNA Synthesis Kit (QuantaBio, Beverly, MA, USA), and the relative expression levels were quantified using qRT-PCR. Real-time qRT-PCR was performed on the StepOne Plus Real-Time PCR System (Applied Biosystems, Foster City, CA, USA) based on SYBR Green fluorescence detection method.

### Fibrosis and inflammation measurement using qRT-PCR and enzyme-linked immunosorbent assay

To evaluate the function of ΔmiR-192 sEV and ΔmiR-432 sEV in fibrosis and inflammation in vitro, we measured the relative mRNA expression levels in inflamed RCF and macrophages, respectively. The procedure timeline is shown in Fig. 4A. Briefly, to measure fibrosis gene expressions, RCFs were cultured in Dulbecco’s modified Eagle’s medium (DMEM; Thermo Fisher Scientific) supplemented with 10% FBS and 1% penicillin-streptomycin. To stimulate fibroblasts, cells were quiesced for 12 hours, treated with sEVs (20 μg/ml) for 12 hours, and then treated with TGF-β (10 ng/ml; Thermo Fisher Scientific) for another 12 hours. Cells were then collected, and RNA was extracted and reverse-transcribed into cDNA for measuring transcript expressions of COL1A1, COL3A1, CTGF, COL1A2, and VIMENTIN. For inflammation analysis, human peripheral blood monocytes (THP-1) were cultured in RPMI 1640 medium (Thermo Fisher Scientific) supplemented with 10% FBS, 1% penicillin-streptomycin, and 1% l-glutamine. Cells were then differentiated into macrophages by 24 hours of incubation with 150 nM phorbol 12-myristate 13-acetate (Sigma-Aldrich) followed by 24 hours of incubation in RPMI medium. Macrophages were then treated with sEVs (20 μg/ml) for 12 hours and activated with TNF-α (20 ng/ml; R&D Systems, Minneapolis, MN, USA) for 12 hours before qRT-PCR. The transcript levels of interleukin-6 (*IL-6*) and *IL-8* were observed. Real-time qRT-PCR was performed as described in the previous section.

### Cell migration assay

Human bone marrow–derived MSCs were cultured in DMEM-F12 supplemented with 10% FBS, 1% penicillin-streptomycin, and FGF-2 (4 ng/ml). Following 12 hours of quiescence in DMEM-F12 without FBS, cells were treated with sEVs (20 μg/ml) for 12 hours. Cells were then detached from the cell culture plate and seeded onto the transwell insert with 8-μm pore (Corning, Corning, NY, USA), and sEVs (20 μg/ml) were added to the bottom well where transwell inserts were placed for 24 hours. Cells that migrated through the porous membrane were collected and stained with the Vybrant DiI Cell-Labeling Solution (Thermo Fisher Scientific) as per the manufacturer’s protocol. The fluorescent level was detected by the Synergy 2 Microplate Reader (Biotek, Winooski, VT, USA).

### Tube formation assay

Rat CECs were cultured in EGM-2 (Lonza, Basel, Switzerland). Following 12 hours of quiescence in EBM-2, cells were treated with sEVs (20 μg/ml) for 12 hours. Cells were then detached, stained with Calcein AM (2 μg/ml; Thermo Fisher Scientific) for 30 min, and seeded (5 × 10^4^ cells/cm^2^) onto the Matrigel-coated plate (4 mg/ml; BD Biosciences, Franklin Lakes, NJ, USA). Six hours after cell seeding, cells were imaged by using a fluorescent microscope (Olympus IX71, Olympus, Tokyo, Japan). Number of branches, total tube length, mesh sizes, and number of meshes were quantified using Fiji software (National Institutes of Health, Bethesda, MD, USA).

### Next-generation sequencing

The sizes, quantities, and integrities of RNA and miRNA isolated from CPCs, sEVs, RCFs, and THP-1 were analyzed by 2100 Bioanalyzer (Agilent Genomics, Santa Clara, CA, USA), and sequencing was conducted by the Emory Yerkes Nonhuman Primate Genomics core (Illumina HiSeq 3000). Small RNAs were aligned, and hits were determined using the Qiagen GeneGlobe console with QIAseq miRNA Quantification tool. Total RNA-seq files were aligned, and gene counts were determined with the STAR aligner in the Illumina BaseSpace app (RNA-Seq Alignment).

### Sequencing data analysis

sEV miRNA sequences from CTR sEVs, ΔmiR-192 sEVs, and ΔmiR-432 sEVs (*n* = 1) were processed using the Illumina BaseSpace Sequence Hub. Adapters were trimmed from the 3′ end and filtered to keep reads 18 to 51 nucleotides in length using the FASTQ Toolkit (version 2.2.5). Then, miRNAs were aligned with the small RNA app (version 1.0.1, homo sapiens hg19 reference, miRbase v21), and mature miRNA counts were exported for downstream analyses. Reads were filtered, keeping miRNAs expressed in at least two samples. Fold change of Δ192 and Δ432 sEV miRNA expression from CTR sEVs was calculated, and miRNAs with |fold change| > 2 were considered. Experimentally validated gene targets (≥3 experiments) of miRNAs were determined using miRTarBase (mirtarbase.cuhk.edu.cn/).

For the recipient cell total RNA-seq analyses, THP-1 and RCF cells were treated with CTR sEV, Δ-miR192 sEV, and ΔmiR-432 sEVs (*n* = 3), and total RNA was sequenced. THP-1 and RCF sequences were aligned with the RNA-Seq Alignment app (version 1.1.0) using the homo sapiens hg19 and rattus norvegicus rn5 references, respectively. One sample from THP treated with ΔmiR-432 sEVs and one sample from RCF treated with Δ-miR192 sEVs were determined to be outliers by multidimensional scaling plots and excluded in subsequent analysis. Genes with zero counts for all samples were removed, filtered with the HTSFilter function (version 4.1) (*43*), and normalized using edgeR’s trimmed means of M method. Plots were produced using counts per million (cpm) values. DEGs were determined with exact test (|fold change| > 1.5 and *P* < 0.05). Pathway analysis of DEGs was performed using Metascape (*44*).

### Cardiac IR model

All animal experiments in this study were reviewed and approved by the Institutional Animal Care and Use Committee at the Emory University (protocol number 201800022). Studies were conducted under a randomized and double-blinded manner. The surgeries were conducted as previously described (*20*). Briefly, male adolescent (6-week-old) Sprague-Dawley rats (Charles River Laboratories, Wilmington, MA, USA) were anesthetized with isoflurane (1 to 3% inhaled) by tracheal intubation, and the chest skin area of each rat was sterilized by serial swabbing with 70% ethanol and betadine. The heart was exposed by incision 1- to 2-cm skin and separation of the ribs. MI was achieved by ligation of the left anterior descending coronary artery with 4-0 silk suture for 30 min, and the suture was removed for reperfusion of the blood. The chests and skin were closed using 4-0 silk suture and 5-0 ethilon suture, respectively. Two weeks after surgery, rats were divided into five groups including Sham, PBS (as control), CTR sEV, ΔmiR-192 sEV, and ΔmiR-432 sEV. For three sEV injection groups, sEVs (5 μg/kg) in 100 μl of PBS were directly injected to the cyanotic ischemic zone through ultrasound image guidance (Vevo 2100; Fujifilm VisualSonics, Toronto, Canada). EF and FS of the left ventricle were monitored using Vevo 2100 ultrasound machine before surgery and 0, 7, 14, and 28 days after surgery. sEV retention was measured using IVIS Spectrum Series (PerkinElmer, Waltham, MA, USA) on days 0, 7, and 14 after DiR-labeled sEV injection, and the average radiant efficiency from the heart was quantified.

### Flow cytometry analysis for quantifying stem cell mobilization

To evaluate stem cell mobilization from bone marrow into the blood circulation by sEV injection, Sprague-Dawley rats that received IR surgery were sacrificed on 7 days after sEV injection and blood was collected from heart. PBMCs were isolated by Ficoll-Paque plus (GE Healthcare, Pittsburgh, PA, USA) gradient centrifugation. Twenty million PBMCs were incubated with allophycocyanin (APC)–conjugated anti-rat CD45 antibody (Miltenyi Biotec, North Rhine-Westphalia, Germany) and fluorescein isothiocyanate (FITC)–conjugated anti-rat CD90 antibody (Abcam, Cambridge, UK). MSC fractions in total PBMCs (*n* = 3) were quantitatively analyzed using Aurora Full Spectrum Cytometry (Cytek, CA, USA) and FlowJo software (BD Biosciences).

### Histological analysis

After 7 and 28 days of ischemic injury, rats were sacrificed, and hearts were harvested for histological analyses. Hearts were fixed with 10% normal formalin, cryoprotected in 30% sucrose, and embedded in optimal cutting temperature (OCT) medium (Thermo Fisher Scientific). OCT sample blocks were cut into 8-μm-thick sections, which were then stained with picrosirius red (Abcam) to measure fibrosis. To measure angiogenesis and cardiac hypertrophy, tissue sections were stained with fluorescein-conjugated IB4 (1:25 dilution) (Vector Laboratories) and rhodamine-conjugated WGA (1:200 dilution) (Vector Laboratories, RL10225), respectively. To measure the necrotic inflammatory responses in day 7, tissue sample sections were stained with FITC-conjugated anti–TNF-α antibody (1:200 dilution) (Bioss Antibodies, bs-10802R-FITC) and anti-ROS antibody (1:500 dilution) (Thermo Fisher Scientific, MA5-26760) with AF-647 secondary antibody (1:200 dilution) (Thermo Fisher Scientific, A-21235). All stained slides were then imaged using a Hamamatsu Nanozoomer 2.0HT slide scanner.

### Statistical analysis

All statistical analyses were performed using GraphPad Prism 9 software (GraphPad, CA, USA). Data are shown as the mean ± SD unless otherwise stated. Unpaired Student’s *t* test and one-way analysis of variance (ANOVA) were applied, and *P* values of <0.05, 0.01, 0.001, and 0.0001 were considered significant in all tests.

## Supplementary Materials

### This PDF file includes:

Figs. S1 to S6 Tables S1 and S2 References

## Notes

View/request a protocol for this paper from *Bio-protocol*.
