# Supplementary material for: Knockdown of deleterious miRNA in progenitor cell–derived small extracellular vesicles enhances tissue repair in myocardial infarction
Source: Sci Adv. 2023 Mar 3;9(9):eabo4616. doi: 10.1126/sciadv.abo4616 (PMC9984177; doi:10.1126/sciadv.abo4616)
Supplement: Supplementary file 1 — Figs. S1 to S6 Tables S1 and S2 References [file sciadv.abo4616_sm.pdf]

Science Advances
AAAS

Supplementary Materials for

Knockdown of deleterious miRNA in progenitor cell-derived small extracellular vesicles enhances tissue repair in myocardial infarction

Hyun-Ji Park *et al.*

Corresponding author: Michael E. Davis, michael.davis@bme.gatech.edu

Sci. Adv. **9**, eabo4616 (2023)
DOI: 10.1126/sciadv.abo4616

This PDF file includes:

Figs. S1 to S6
Tables S1 and S2
References

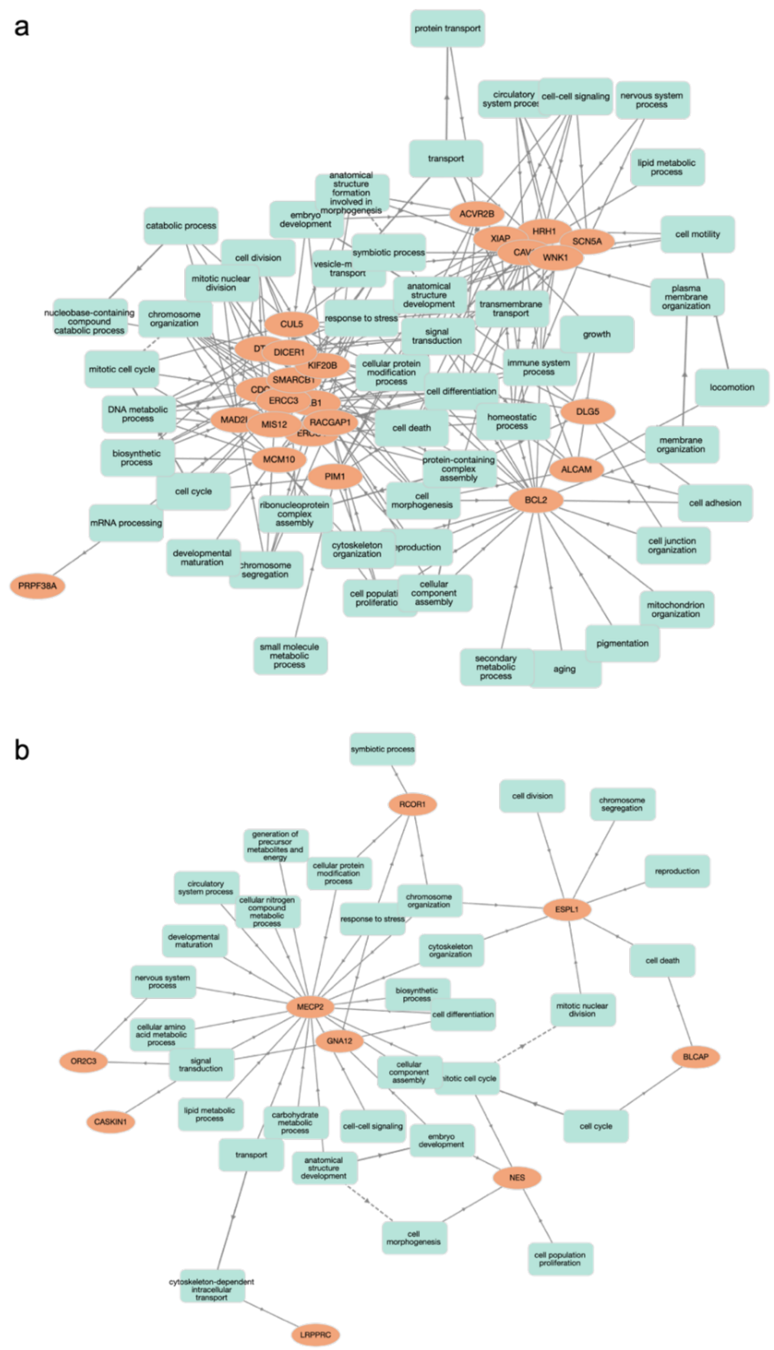

Supplementary Fig. S1. Interactive target gene annotation analyses of GO biological processes showed the connections between miRNA gene targets and biological processes. a, 25 targets of miR-192-5p and **b,** 9 targets of miR-432-5p that are validated with more than three assays. Interactive maps of target gene-GO terms were generated from GOnet web-based tool for interactive Gene Ontology analysis (https://tools.dice-database.org)[45].

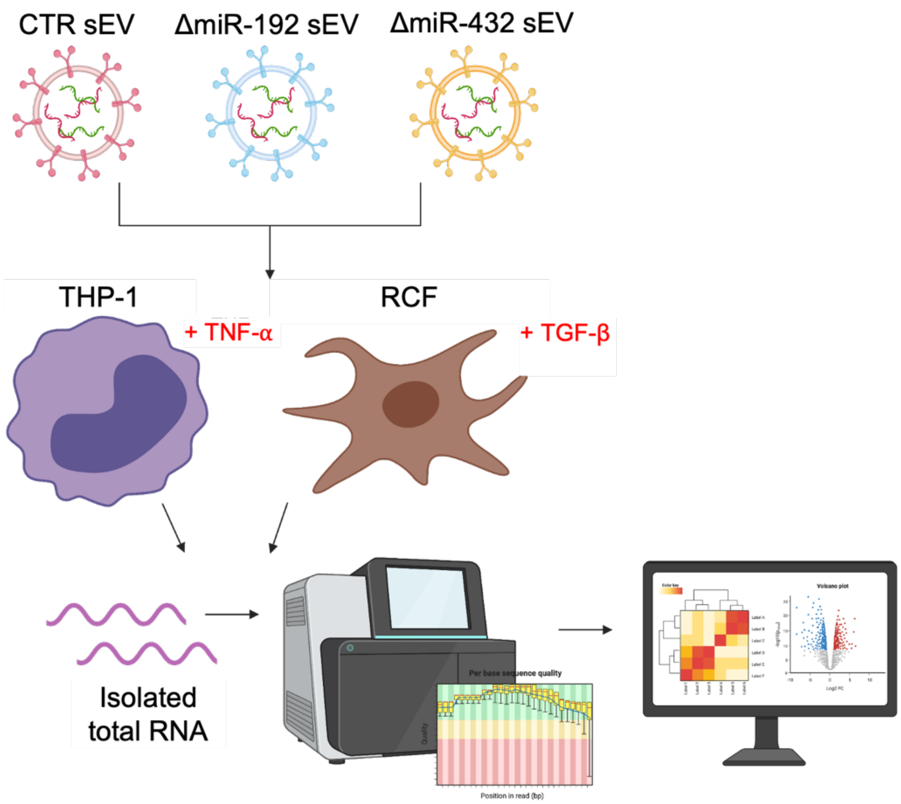

Supplementary Fig. S2. Schematic illustrations showed the process of recipient cells (THP-1 and RCF) RNA sequencing after various sEV treatment.

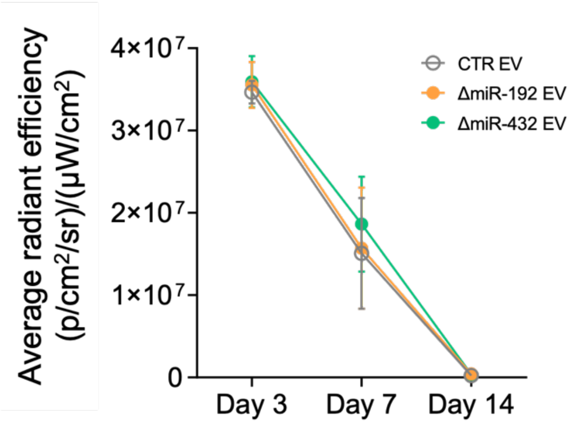

Supplementary Fig. S3. Time-course analysis determined the average radiant efficiency of sEVs in the heart 3-, 7-, and 14-days post sEV injection (n=4).

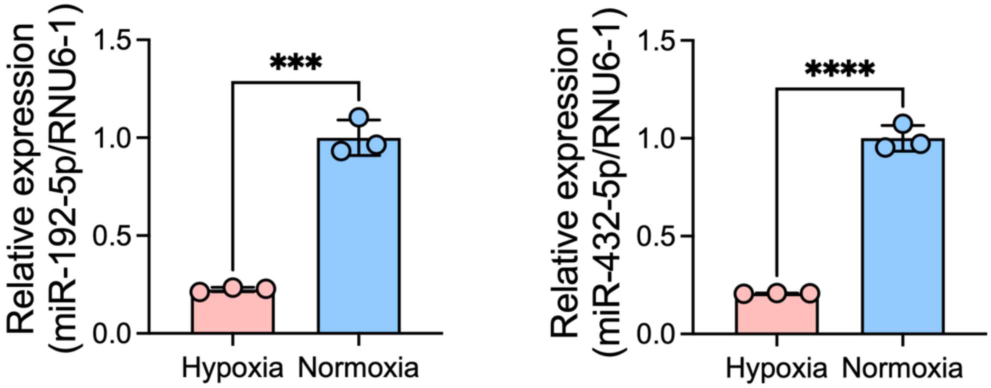

Supplementary Fig. S4. Relative miRNA expressions in CPC sEVs. CPCs were cultured under different oxygen concentration for 24 hours before serum depletion. sEVs were collected after 24 hours of serum depletion. ***p<0.001 and ****p<0.0001.

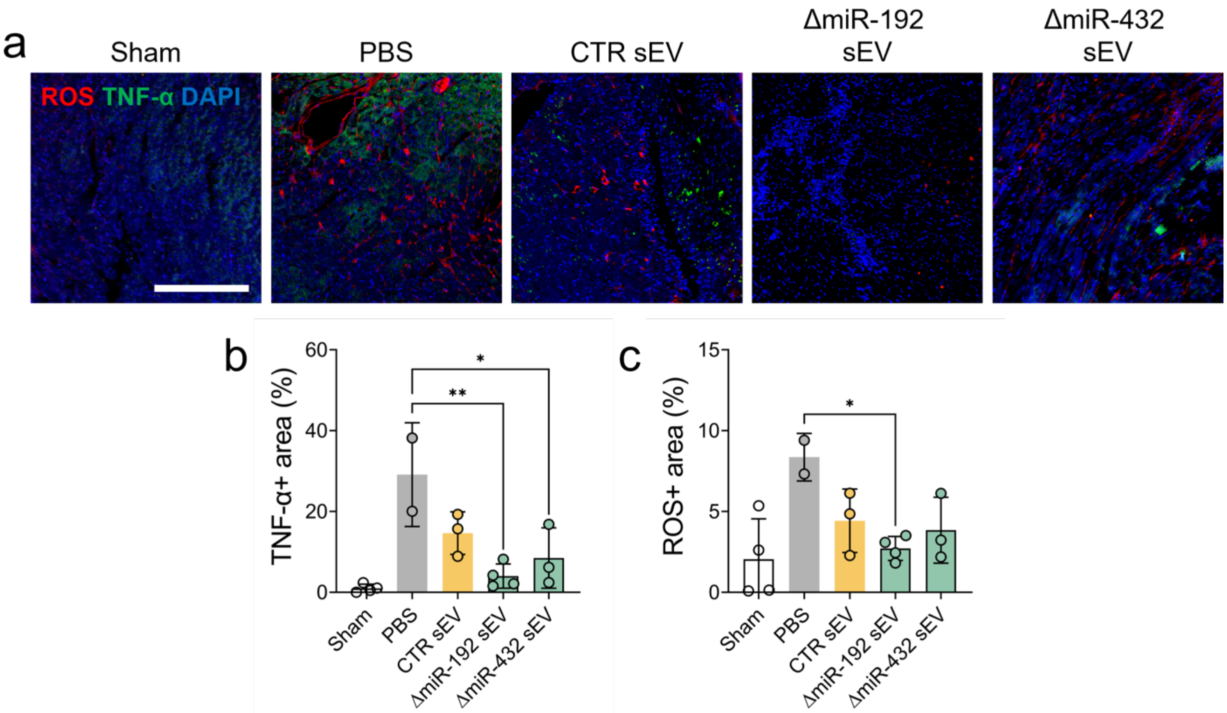

Supplementary Fig. S5. sEV administration showed anti-inflammatory responses in cardiac IR rats 7 days post-treatment. a, Immunohistochemical staining of ischemic hearts to detect TNF-α (green) and ROS (red) expressions. Scale bar indicates 200 µm. **b,** TNF-α positive area and **c,** ROS positive area measured from stained images. *$p<0.05$ and **$p<0.01$.

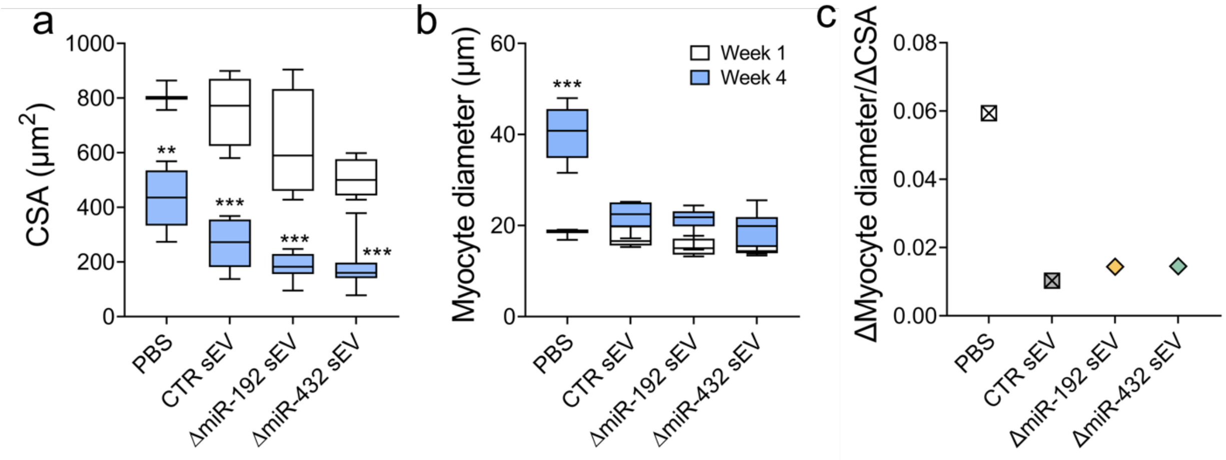

Supplementary Fig. S6. Morphological changes of myoblasts were observed in cardiac IR rats 1- and 4-weeks post treatment. a, CSA and **b,** myocyte diameter changes between week 1 and week 4 measured from WGA-stained images. **p<0.01 and ***p<0.001. **c,** Average ratio of changes of myocyte diameter and changes of CSA between week 1 and week 4.

Supplementary Table S1. 50 VIP miRNAs with high relativity with angiogenesis, fibrosis, and left ventricular EF in cardiac IR rat after exosome therapy.

Agarwal et al. (Ref 20)				Trac et al. (Ref 21)			
1	hsa-miR-874-3p	26	hsa-miR-23a-3p	1	hsa-miR-323a-3p	26	hsa-miR-146a-5p
2	hsa-miR-378f	27	hsa-miR-3158-5p	2	hsa-miR-335-3p	27	hsa-miR-125b-1-3p
3	hsa-miR-3909	28	hsa-miR-4637	3	hsa-miR-328-3p	28	hsa-miR-27a-3p
4	hsa-miR-4477a	29	hsa-miR-185-5p	4	hsa-miR-204-3p	29	hsa-miR-145-5p
5	hsa-miR-4520-5p	30	hsa-miR-4670-5p	5	hsa-miR-654-5p	30	hsa-miR-382-5p
6	hsa-miR-362-5p	31	hsa-miR-4758-5p	6	hsa-miR-16-5p	31	hsa-miR-127-3p
7	hsa-miR-718	32	hsa-miR-3144-3p	7	hsa-miR-210-3p	32	hsa-miR-148a-3p
8	hsa-miR-548am-3p	33	hsa-miR-4704-5p	8	hsa-miR-27a-5p	33	hsa-miR-769-5p
9	hsa-miR-4760-5p	34	hsa-miR-3199	9	hsa-miR-28-3p	34	hsa-miR-409-3p
10	hsa-miR-590-3p	35	hsa-miR-2909	10	hsa-miR-92b-3p	35	hsa-miR-151a-5p
11	hsa-miR-3186-3p	36	hsa-miR-633	11	hsa-miR-378a-3p	36	hsa-miR-671-3p
12	hsa-miR-15a-5p	37	hsa-miR-3163	12	hsa-miR-320c	37	hsa-let-7c-5p
13	hsa-miR-513b-5p	38	hsa-miR-545-3p	13	hsa-miR-486-5p	38	hsa-miR-432-5p
14	hsa-miR-4797-3p	39	hsa-miR-192-5p	14	hsa-miR-23b-3p	39	hsa-miR-151a-3p
15	hsa-miR-619-3p	40	hsa-miR-4755-3p	15	hsa-let-7d-3p	40	hsa-miR-221-3p
16	hsa-miR-4310	41	hsa-miR-432-5p	16	hsa-miR-134-5p	41	hsa-miR-25-3p
17	hsa-miR-4725-3p	42	hsa-miR-3136-5p	17	hsa-miR-34a-5p	42	hsa-miR-543
18	hsa-miR-4797-5p	43	hsa-miR-548e-3p	18	hsa-miR-423-5p	43	hsa-miR-100-5p
19	hsa-miR-4638-3p	44	hsa-miR-3189-3p	19	hsa-miR-22-3p	44	hsa-miR-192-5p
20	hsa-miR-339-3p	45	hsa-miR-1973	20	hsa-miR-146b-5p	45	hsa-let-7e-5p
21	hsa-miR-4736	46	hsa-miR-335-5p	21	hsa-miR-30a-3p	46	hsa-miR-532-5p
22	hsa-miR-1270	47	hsa-miR-3617-5p	22	hsa-miR-320a	47	hsa-miR-937-3p
23	hsa-miR-766-3p	48	hsa-miR-154-5p	23	hsa-miR-483-5p	48	hsa-let-7i-5p
24	hsa-miR-4275	49	hsa-miR-1288-3p	24	hsa-miR-99b-5p	49	hsa-miR-941
25	hsa-miR-3125	50	hsa-miR-544a	25	hsa-miR-370-3p	50	hsa-miR-99a-5p

Supplementary Table S2. Target genes of miR-192-5p and miR-432-5p validated with at least three assays.

miRNA	Target	Number of assays to validate target
hsa-miR-192-5p	ALCAM	5
	CDC7	5
	CUL5	5
	ERCC3	5
	LMNB2	5
	MAD2L1	5
	PIM1	5
	ERCC4	4
	RB1	4
	BCL2	4
	DLG5	4
	DTL	4
	HRH1	4
	MCM10	4
	MIS12	4
	KIF20B	4
	PRPF38A	4
	RACGAP1	4
	SEPT10	4
	SMARCB1	4
	WNK1	4
	XIAP	4
	DICER1	4
	ACVR2B	3
	CAV1	3
	SCN5A	3
hsa-miR-432-5p	MECP2	3
	RCOR1	3
	NES	3

REFERENCES AND NOTES

1. World Health Organization, Cardiovascular diseases (CVDs) (2020); www.who.int/news-room/fact-sheets/detail/cardiovascular-diseases-(cvds).
2. O. J. Mechanic, M. Gavin, S. A. Grossman, *Acute Myocardial Infarction* (StatPearls Publishing, 2021).
3. B. E. Strauer, G. Steinhoff, 10 years of intracoronary and intramyocardial bone marrow stem cell therapy of the heart: From the methodological origin to clinical practice. *J. Am. Coll. Cardiol.* **58**, 1095–1104 (2011).
4. V. Karantalis, V. Y. Suncion-Loescher, L. Bagno, S. Golpanian, A. Wolf, C. Sanina, C. Premer, A. J. Kanelidis, F. McCall, B. Wang, W. Balkan, J. Rodriguez, M. Rosado, A. Morales, K. Hatzistergos, M. Natsumeda, I. Margitich, I. H. Schulman, S. A. Gomes, M. Mushtaq, D. L. DiFede, J. E. Fishman, P. Pattany, J. P. Zambrano, A. W. Heldman, J. M. Hare, Synergistic effects of combined cell therapy for chronic ischemic cardiomyopathy. *J. Am. Coll. Cardiol.* **66**, 1990–1999 (2015).
5. J. Kandala, G. A. Upadhyay, E. Pokushalov, S. Wu, D. E. Drachman, J. P. Singh, Meta-analysis of stem cell therapy in chronic ischemic cardiomyopathy. *Am. J. Cardiol.* **112**, 217–225 (2013).
6. A. E.-S. Shafei, M. A. Ali, H. G. Ghanem, A. I. Shehata, A. A. Abdelgawad, H. R. Handal, K. A. Talaat, A. E. Ashaal, A. S. El-Shal, Mesenchymal stem cell therapy: A promising cell-based therapy for treatment of myocardial infarction. *J. Gene Med.* **19**, e2995 (2017).
7. A. J. Kanelidis, C. Premer, J. Lopez, W. Balkan, J. M. Hare, Route of delivery modulates the efficacy of mesenchymal stem cell therapy for myocardial infarction: A meta-analysis of preclinical studies and clinical trials. *Circ. Res.* **120**, 1139–1150 (2017).
8. R. Bolli, R. D. Mitrani, J. M. Hare, C. J. Pepine, E. C. Perin, J. T. Willerson, J. H. Traverse, T. D. Henry, P. C. Yang, M. P. Murphy, K. L. March, I. H. Schulman, S. Ikram, D. P. Lee, C. O'Brien, J. A. Lima, M. R. Ostovaneh, B. Ambale-Venkatesh, G. Lewis, A. Khan, K. Bacallao, K. Valasaki, B. Longsomboon, A. P. Gee, S. Richman, D. A. Taylor, D. Lai, S. L. Sayre, J. Bettencourt, R. W. Vojvodic, M. L. Cohen, L. Simpson, D. Aguilar, C. Loghin, L. Moyé, R. F. Ebert, B. R. Davis, R. D. Simari; Cardiovascular Cell Therapy Research Network (CCTRN), A phase II study of autologous mesenchymal stromal cells and c-kit positive cardiac cells, alone or in combination, in patients with ischaemic heart failure: The CCTRN CONCERT-HF trial. *Eur. J. Heart Fail.* **23**, 661–674 (2021).
9. R. Bolli, J. M. Hare, K. L. March, C. J. Pepine, J. T. Willerson, E. C. Perin, P. C. Yang, T. D. Henry, J. H. Traverse, R. D. Mitrani, A. Khan, I. Hernandez-Schulman, D. A. Taylor, D. DiFede, J. A. C. Lima, A. Chugh, J. Loughran, R. W. Vojvodic, S. L. Sayre, J. Bettencourt, M. Cohen, L. Moyé, R. F. Ebert, R. D. Simari; Cardiovascular Cell Therapy Research Network (CCTRN), Rationale and design of the CONCERT-HF trial (combination of mesenchymal and c-kit$^+$ cardiac stem cells as regenerative therapy for heart failure). *Circ. Res.* **122**, 1703–1715 (2018).
10. V. Karantalis, D. DiFede, G. Gerstenblith, S. Pham, J. Symes, J. P. Zambrano, J. Fishman, P. Pattany, I. McNiece, J. Conte, S. Schulman, K. Wu, A. Shah, E. Breton, J. Davis-Sproul, R. Schwarz, G. Feigenbaum, M. Mushtaq, V. Y. Suncion, A. C. Lardo, I.

Borrello, A. Mendizabal, T. Z. Karas, J. Byrnes, M. Lowery, A. W. Heldman, J. M. Hare, Autologous mesenchymal stem cells produce concordant improvements in regional function, tissue perfusion, and fibrotic burden when administered to patients undergoing coronary artery bypass grafting: The Prospective Randomized Study of Mesenchymal Stem Cell Therapy in Patients Undergoing Cardiac Surgery (PROMETHEUS) trial. *Circ. Res.* **114**, 1302–1310 (2014).

11. A. W. Heldman, D. L. DiFede, J. E. Fishman, J. P. Zambrano, B. H. Trachtenberg, V. Karantalis, M. Mushtaq, A. R. Williams, V. Y. Suncion, I. K. McNiece, E. Ghersin, V. Soto, G. Lopera, R. Miki, H. Willens, R. Hendel, R. Mitrani, P. Pattany, G. Feigenbaum, B. Oskouei, J. Byrnes, M. H. Lowery, J. Sierra, M. V. Pujol, C. Delgado, P. J. Gonzalez, J. E. Rodriguez, L. L. Bagno, D. Rouy, P. Altman, C. W. P. Foo, J. da Silva, E. Anderson, R. Schwarz, A. Mendizabal, J. M. Hare, Transendocardial mesenchymal stem cells and mononuclear bone marrow cells for ischemic cardiomyopathy: The TAC-HFT randomized trial. *JAMA* **311**, 62–73 (2014).
12. C. L. Mummery, R. P. Davis, J. E. Krieger, Challenges in using stem cells for cardiac repair. *Sci. Transl. Med.* **2**, 27ps17 (2010).
13. M. Korf-Klingebiel, T. Kempf, T. Sauer, E. Brinkmann, P. Fischer, G. P. Meyer, A. Ganser, H. Drexler, K. C. Wollert, Bone marrow cells are a rich source of growth factors and cytokines: Implications for cell therapy trials after myocardial infarction. *Eur. Heart J.* **29**, 2851–2858 (2008).
14. S. Das, M. K. Halushka, Extracellular vesicle microRNA transfer in cardiovascular disease. *Cardiovasc. Pathol.* **24**, 199–206 (2015).
15. P. Saha, S. Sharma, L. Korutla, S. R. Datla, F. Shoja-Taheri, R. Mishra, G. E. Bigham, M. Sarkar, D. Morales, G. Bittle, M. Gunasekaran, C. Ambastha, M. Y. Arfat, D. Li, A. Habertheuer, R. Hu, M. O. Platt, P. Yang, M. E. Davis, P. Vallabhajosyula, S. Kaushal, Circulating exosomes derived from transplanted progenitor cells aid the functional recovery of ischemic myocardium. *Sci. Transl. Med.* **11**, eaau1168 (2019).
16. H. J. Park, K. J. De Jesus Morales, S. Bheri, B. P. Kassouf, M. E. Davis, Bidirectional relationship between cardiac extracellular matrix and cardiac cells in ischemic heart disease. *Stem Cells* **39**, 1650–1659 (2021).
17. S. Bheri, B. P. Kassouf, H. J. Park, J. R. Hoffman, M. E. Davis, Engineering cardiac small extracellular vesicle-derived vehicles with thin-film hydration for customized microRNA loading. *J. Cardiovasc. Dev. Dis.* **8**, 135 (2021).
18. J. O'Brien, H. Hayder, Y. Zayed, C. Peng, Overview of MicroRNA biogenesis, mechanisms of actions, and circulation. *Front. Endocrinol.* **9**, 402 (2018).
19. Y. Song, C. Zhang, J. Zhang, Z. Jiao, N. Dong, G. Wang, Z. Wang, L. Wang, Localized injection of miRNA-21-enriched extracellular vesicles effectively restores cardiac function after myocardial infarction. *Theranostics* **9**, 2346–2360 (2019).
20. U. Agarwal, A. George, S. Bhutani, S. Ghosh-Choudhary, J. T. Maxwell, M. E. Brown, Y. Mehta, M. O. Platt, Y. Liang, S. Sahoo, M. E. Davis, Experimental, systems, and computational approaches to understanding the MicroRNA-mediated reparative potential of cardiac progenitor cell-derived exosomes from pediatric patients. *Circ. Res.* **120**, 701–712 (2017).

21. D. Trac, J. R. Hoffman, S. Bheri, J. T. Maxwell, M. O. Platt, M. E. Davis, Predicting functional responses of progenitor cell exosome potential with computational modeling. *Stem Cells Transl. Med.* **8**, 1212–1221 (2019).
22. J. Mayourian, D. K. Ceholski, P. A. Gorski, P. Mathiyalagan, J. F. Murphy, S. I. Salazar, F. Stillitano, J. M. Hare, S. Sahoo, R. J. Hajjar, K. D. Costa, Exosomal microRNA-21-5p mediates mesenchymal stem cell paracrine effects on human cardiac tissue contractility. *Circ. Res.* **122**, 933–944 (2018).
23. H. Khalil, O. Kanisicak, V. Prasad, R. N. Correll, X. Fu, T. Schips, R. J. Vagnozzi, R. Liu, T. Huynh, S. J. Lee, J. Karch, J. D. Molkentin, Fibroblast-specific TGF-β–Smad2/3 signaling underlies cardiac fibrosis. *J. Clin. Invest.* **127**, 3770–3783 (2017).
24. S. Ma, N. Xie, W. Li, B. Yuan, Y. Shi, Y. Wang, Immunobiology of mesenchymal stem cells. *Cell Death Differ.* **21**, 216–225 (2014).
25. F. Marofi, K. I. Alexandrovna, R. Margiana, M. Bahramali, W. Suksatan, W. K. Abdelbasset, S. Chupradit, M. Nasimi, M. S. Maashi, MSCs and their exosomes: A rapidly evolving approach in the context of cutaneous wounds therapy. *Stem Cell Res. Ther.* **12**, 597 (2021).
26. A. Sica, A. Mantovani, Macrophage plasticity and polarization: In vivo veritas. *J. Clin. Invest.* **122**, 787–795 (2012).
27. Y. Zhang, R. Huang, W. Zhou, Q. Zhao, Z. Lu, miR-192-5p mediates hypoxia/reoxygenation-induced apoptosis in H9c2 cardiomyocytes via targeting of FABP3. *J. Biochem. Mol. Toxicol.* **31**, e21873 (2017).
28. F. Sun, W. Yuan, H. Wu, G. Chen, Y. Sun, L. Yuan, W. Zhang, M. Lei, LncRNA KCNQ1OT1 attenuates sepsis-induced myocardial injury via regulating miR-192-5p/XIAP axis. *Exp. Biol. Med. (Maywood)* **245**, 620–630 (2020).
29. J. Chen, J. Wang, H. Li, S. Wang, X. Xiang, D. Zhang, p53 activates miR-192-5p to mediate vancomycin induced AKI. *Sci. Rep.* **6**, 38868 (2016).
30. M. Puppo, G. Bucci, M. Rossi, M. Giovarelli, D. Bordo, A. Moshiri, F. Gorlero, R. Gherzi, P. Briata, miRNA-mediated KHSRP silencing rewires distinct post-transcriptional programs during TGF-beta-Induced epithelial-to-mesenchymal transition. *Cell Rep.* **16**, 967–978 (2016).
31. L. Fang, A. H. Ellims, X. L. Moore, D. A. White, A. J. Taylor, J. Chin-Dusting, A. M. Dart, Circulating microRNAs as biomarkers for diffuse myocardial fibrosis in patients with hypertrophic cardiomyopathy. *J. Transl. Med.* **13**, 314 (2015).
32. C. Li, G. Zhou, J. Feng, J. Zhang, L. Hou, Z. Cheng, Upregulation of lncRNA VDR/CASC15 induced by facilitates cardiac hypertrophy through modulating miR-432-5p/TLR4 axis. *Biochem. Biophys. Res. Commun.* **503**, 2407–2414 (2018).
33. M. Sancho-Albero, N. Navascués, G. Mendoza, V. Sebastián, M. Arruebo, P. Martín-Duque, J. Santamaría, Exosome origin determines cell targeting and the transfer of therapeutic nanoparticles towards target cells. *J Nanobiotechnol.* **17**, 16 (2019).
34. M. A. Serrano-Rosa, E. León-Zarceño, C. Giglio, S. Boix-Vilella, A. Moreno-Tenas, L. Pamies-Aubalat, V. Arrarte, Psychological state after an acute coronary syndrome: Impact of physical limitations. *Int. J. Environ. Res. Public Health* **18**, 6473 (2021).
35. H. Blaser, C. Dostert, T. W. Mak, D. Brenner, TNF and ROS crosstalk in Inflammation. *Trends Cell Biol.* **26**, 249–261 (2016).

36. D.-W. Zhang, J. Shao, J. Lin, N. Zhang, B. J. Lu, S. C. Lin, M. Q. Dong, J. Han, RIP3, an energy metabolism regulator that switches TNF-induced cell death from apoptosis to necrosis. *Science* **325**, 332–336 (2009).
37. A. M. Gerdes, T. Onodera, X. Wang, S. A. McCune, Myocyte remodeling during the progression to failure in rats with hypertension. *Hypertension* **28**, 609–614 (1996).
38. A. Heidersbach, C. Saxby, K. Carver-Moore, Y. Huang, Y.-S. Ang, P. J. de Jong, K. N. Ivey, D. Srivastava, microRNA-1 regulates sarcomere formation and suppresses smooth muscle gene expression in the mammalian heart. *eLife* **2**, e01323 (2013).
39. Y. Zhao, J. F. Ransom, A. Li, V. Vedantham, M. von Drehle, A. N. Muth, T. Tsuchihashi, M. T. McManus, R. J. Schwartz, D. Srivastava, Dysregulation of cardiogenesis, cardiac conduction, and cell cycle in mice lacking miRNA-1-2. *Cell* **129**, 303–317 (2007).
40. Y. Wei, S. Peng, M. Wu, R. Sachidanandam, Z. Tu, S. Zhang, C. Falce, E. A. Sobie, D. Lebeche, Y. Zhao, Multifaceted roles of miR-1s in repressing the fetal gene program in the heart. *Cell Res.* **24**, 278–292 (2014).
41. D. Rokad, H. Jin, V. Anantharam, A. Kanthasamy, A. G. Kanthasamy, Exosomes as mediators of chemical-induced toxicity. *Curr. Environ. Health Rep.* **6**, 73–79 (2019).
42. K. M. French, M. E. Davis, Isolation and expansion of c-kit-positive cardiac progenitor cells by magnetic cell sorting. *Methods Mol. Biol.* **1181**, 39–50 (2014).
43. A. Rau, M. Gallopin, G. Celeux, F. Jaffrezic, Data-based filtering for replicated high-throughput transcriptome sequencing experiments. *Bioinformatics* **29**, 2146–2152 (2013).
44. Y. Zhou, B. Zhou, L. Pache, M. Chang, A. H. Khodabakhshi, O. Tanaseichuk, C. Benner, S. K. Chanda, Metascape provides a biologist-oriented resource for the analysis of systems-level datasets. *Nat. Commun.* **10**, 1523 (2019).
45. M. Pomaznoy, B. Ha, B. Peters, GOnet: A tool for interactive gene ontology analysis. *BMC Bioinformatics* **19**, 470 (2018).
